# Physical activity and exercise in chronic kidney disease: consensus statements from the Physical Exercise Working Group of the Italian Society of Nephrology

**DOI:** 10.1007/s40620-024-02049-9

**Published:** 2024-09-13

**Authors:** Yuri Battaglia, Federica Baciga, Francesca Bulighin, Maria Amicone, Giovanni Mosconi, Alda Storari, Rachele Brugnano, Marco Pozzato, Daria Motta, Claudia D’alessandro, Claudia Torino, Francesca Mallamaci, Adamasco Cupisti, Filippo Aucella, Alessandro Capitanini, Working Group of Physical Exercise of Italian Society of Nephrology

**Affiliations:** 1 Department of Medicine, University of Verona, Verona, Italy; 2 Nephrology and Dialysis Unit, Pederzoli Hospital, Via Monte Baldo, 24, Peschiera del Garda, 37019 Verona, Italy; 3 Department of Public Health, Chair of Nephrology, University of Naples Federico II, Naples, Italy; 4 Nephrology and Dialysis Unit, Morgagni-Pierantoni Hospital, Forlì, Italy; 5 Nephrology and Dialysis Unit, University of Ferrara, Ferrara, Italy; 6 Nephrology and Dialysis Unit, S. Maria Della Misericordia Hospital, Perugia, Italy; 7 Nephrology and Dialysis Unit, S. Giovanni Bosco Hospital, University of Turin, Turin, Italy; 8 Nephrology and Dialysis Unit, Martini Hospital, ASL Città Di Torino, Turin, Italy; 9 Department of Clinical and Experimental Medicine, University of Pisa, Pisa, Italy; 10 Institute of Clinical Physiology, National Research Council, Reggio Calabria, Italy; 11 Nephrology and Dialysis Unit, Bianchi-Melacrino-Morelli Hospital, Reggio Calabria, Italy; 12 Nephrology and Dialysis Unit, Casa Solievo Della Sofferenza, San Giovanni Rotondo Foggia, Italy; 13 Nephrology and Dialysis Unit, San Jacopo Hospital, Pistoia, Italy

**Keywords:** Exercise, Renal rehabilitation, Barriers, Guidelines, Sarcopenia

## Abstract

The Italian Society of Nephrology has tasked its Working Group on Physical Exercise with developing a consensus statement document on physical activity and exercise in patients with chronic kidney disease (CKD). This paper consists of 16 points that were discussed, and approved using the mini-Delphi method by 15 members of the working group. Each statement is based on a comprehensive review of the literature, clinical experience, and expert opinions. Overall, the statements affirm that regular physical activity and exercise training offer numerous benefits to CKD patients, including improved physical function, enhanced cardiometabolic and neuromuscular function, cognitive benefits, and an overall improvement in quality of life. Furthermore, exercise may provide nephroprotection and reduce mortality. These advantages are observed across all CKD stages, whether on conservative therapy or kidney replacement therapy (hemodialysis or peritoneal dialysis), and in kidney transplant recipients. Moreover, when physical activity and exercise training are implemented with appropriate precautions, they are safe in CKD patients. Gradual physical activity and customized exercise programs should be tailored to the patient's exercise tolerance, potentially enhancing compliance. Clinicians are encouraged to use a series of questionnaires and tests to assess the patient's level of physical activity and performance. However, exercise and physical activity are poorly implemented in clinical practice due to many barriers related to patients and healthcare staff. Overcoming these barriers requires the proactive role of the nephrologists, who should actively incorporate exercise training and promote physical activity within routine care plans. Adopting a multidisciplinary team approach, which includes nephrologists, nurses, exercise professionals, and dietitians, is crucial for providing comprehensive rehabilitation for CKD patients. Integrating new technologies and remote check ups could further enhance the effectiveness of these interventions.

**Graphical abstract:**

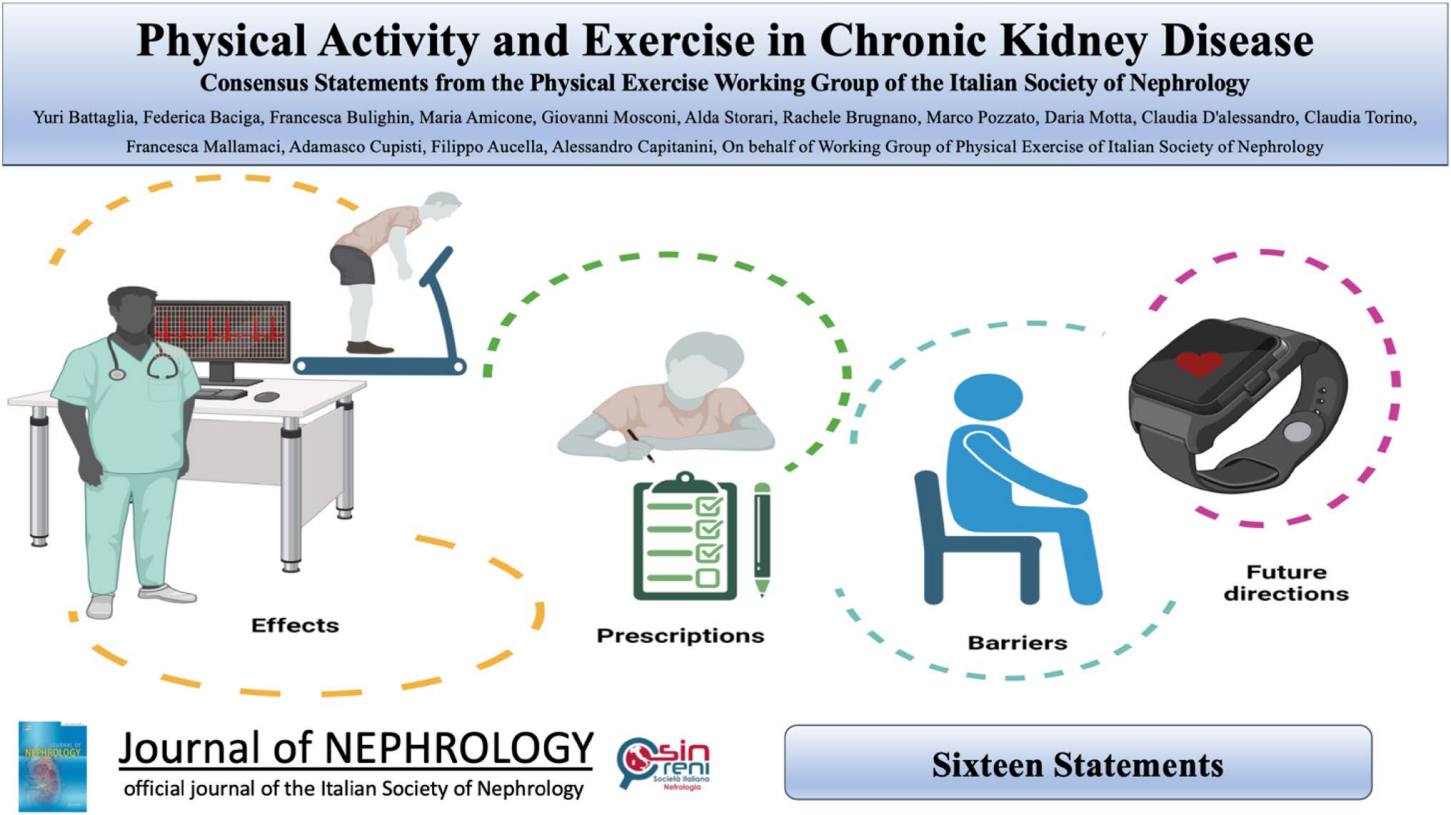

## Introduction

The first recommendations in the field of physical activity and exercise in patients with chronic kidney diseases (CKD) were published in 2005 and were included in the Kidney Disease Outcomes Quality Initiative (KDOQI) guidelines only for hemodialysis patients [1]. These guidelines state that nephrologists and hemodialysis staff should encourage all hemodialysis patients to maintain or increase their physical activity level, with a recommended goal of 30 min of moderate physical activity most days of the week.

In 2012, the Kidney Disease: Improving Global Outcomes (KDIGO) guidelines [2] expanded the recommendation of physical activity to patients with CKD stages 3–5, suggesting at least 30 min 5 times a week based on the patient’s cardiovascular status and tolerance. However, these guidelines are adapted from the general population or other chronic conditions with more robust evidence than in the CKD setting. It is worth noting that patients with CKD differ from the general population or those with other chronic conditions in terms of physical capacity and comorbidities [3, 4], exhibiting wide heterogeneity in both underlying kidney diseases and different CKD stages. The presence of CKD itself, along with other comorbidities (e.g., physical inactivity, arterial hypertension, dyslipidemia, obesity, and diabetes), contributes to a high cardiovascular disease (CVD) risk in CKD patients [5]. Nonetheless, in the Consensus Statement from EXercise Prescription in Everyday practice and Rehabilitative Training (EXPERT) Working Group, obesity, dyslipidemia, diabetes, and hypertension, but not CKD, were considered for exercise prescription in patients with different CVD risk factor combinations [6]. Similarly, the latest World Health Organization (WHO) Guidelines released in 2020 [7] are targeted at adults or older individuals with chronic conditions, including hypertension, diabetes, cancer, and HIV, but kidney disease is not specifically mentioned.

The first guidelines on exercise specifically for CKD populations, categorizing patients into conservative treatment, dialysis, or kidney transplant recipients (KTRs), were developed by the UK Kidney Research Consortium Clinical Study Group for exercise and lifestyle [8] in 2020. Similarly, the recommendations on physical activity in the updated KDIGO 2024 guidelines, with grade 1D evidence, were extended to all patients with CKD, and suggest advising patients to engage in moderate-intensity physical activity for at least 150 min per week, based on their cardiovascular and physical tolerance [9].

Nonetheless, it is crucial to emphasize that these recommendations are concise statements within guidelines lacking clinical practice suggestions. Firstly, as revealed by a scoping review of current recommendations on physical activity and exercise in dialysis patients, there are discrepancies in the terms used for exercise, physical activity, frequency, duration, intensity, and type [10]. Additionally, physical activity and exercise prescriptions should be tailored to each patient, considering factors such as physical function, comorbidities, space availability, and time to ensure that physical exercise is adequate, safe, and feasible [11]. Although further multicenter studies are needed to improve our knowledge in this field, the Italian Society of Nephrology has tasked the Working Group on Physical Exercise to develop a consensus statement document regarding physical activity and exercise in patients affected by CKD.

## Methods

A comprehensive literature review was conducted, primarily focusing on original research due to the scarcity of systematic reviews, meta-analyses, and randomized controlled trials specifically addressing physical activity and exercise in CKD. Available guidelines and clinical practice recommendations were also reviewed.

**Fig. 1:**
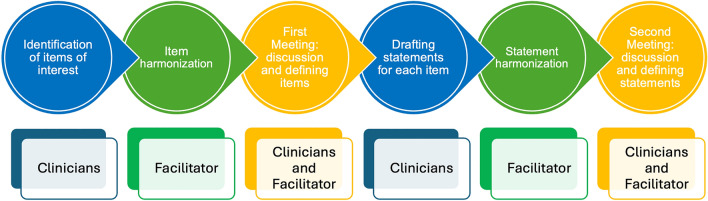
Project flowchart

Using the Estimate-Talk-Estimate (ETE) method, or “mini-Delphi” [12, 13], 20 points of interest were initially proposed by 15 experts and then harmonized with a facilitator, resulting in 16 final items. One statement was generated for each item based on an in-depth review of the evidence, clinical practice experience, and expert opinion. During a plenary session, the 16 final statements were thoroughly discussed and unanimously approved with a strong level of agreement among members of the Working Group. The workflow of the project is illustrated in Fig. 1.

## Summary of statements

1. "Physical activity" and "exercise" have distinct meanings and should be used accordingly.
2. Physical inactivity is prevalent in CKD patients and represents a modifiable risk factor for increased mortality and morbidity, as well as for reduced quality of life
3. Physical activity and exercise significantly benefit CKD patients, particularly in improving physical function, cardiorespiratory capacity, muscle strength, and overall quality of life.
4. Physical exercise is safe in CKD patients when appropriately performed.
5. Physical function assessment can be performed routinely through physical performance tests and patient-focused questionnaires.
6. Monitoring physical activity levels using self-report instruments or objective measures is advisable to counteract sedentary behavior.
7. Protein restriction does not blunt or prevent the favorable effects of exercise on muscle strength and mass in CKD patients.
8. Dietary supplements combined with exercise are an effective strategy for preventing sarcopenia or protein-energy wasting in CKD patients, including those on dialysis.
9. Barriers to physical activity in the CKD population are well known: each nephrology and dialysis unit needs to identify its center-specific barriers and establish a detailed plan for overcoming them.
10. It is crucial to personalize physical activity prescriptions, which can be provided by a nephrologist, while in the presence of functional limitations, a physiotherapist or sports medicine physician consultation is generally needed.
11. Exercise training for CKD patients can be undertaken alone or in groups, at home, in a dialysis unit, or in a sports facility.
12. Exercise training for CKD patients should include aerobic and resistance exercises.
13. Exercise programs should be carried out regularly for at least 12 weeks to be effective.
14. Physical activity and exercise programs should begin at low intensity and progress gradually according to the patient's tolerance.
15. In everyday clinical practice, renal rehabilitation should be provided by a healthcare team (nephrologists, nurses, dietitians, and exercise professionals) and, ideally, reimbursed by the national healthcare system.

16. New technologies, such as interactive social media creation and virtual reality gaming, could improve CKD patients' adherence to exercise implementation.

## "Physical activity" and "exercise" have distinct meanings and should be used accordingly


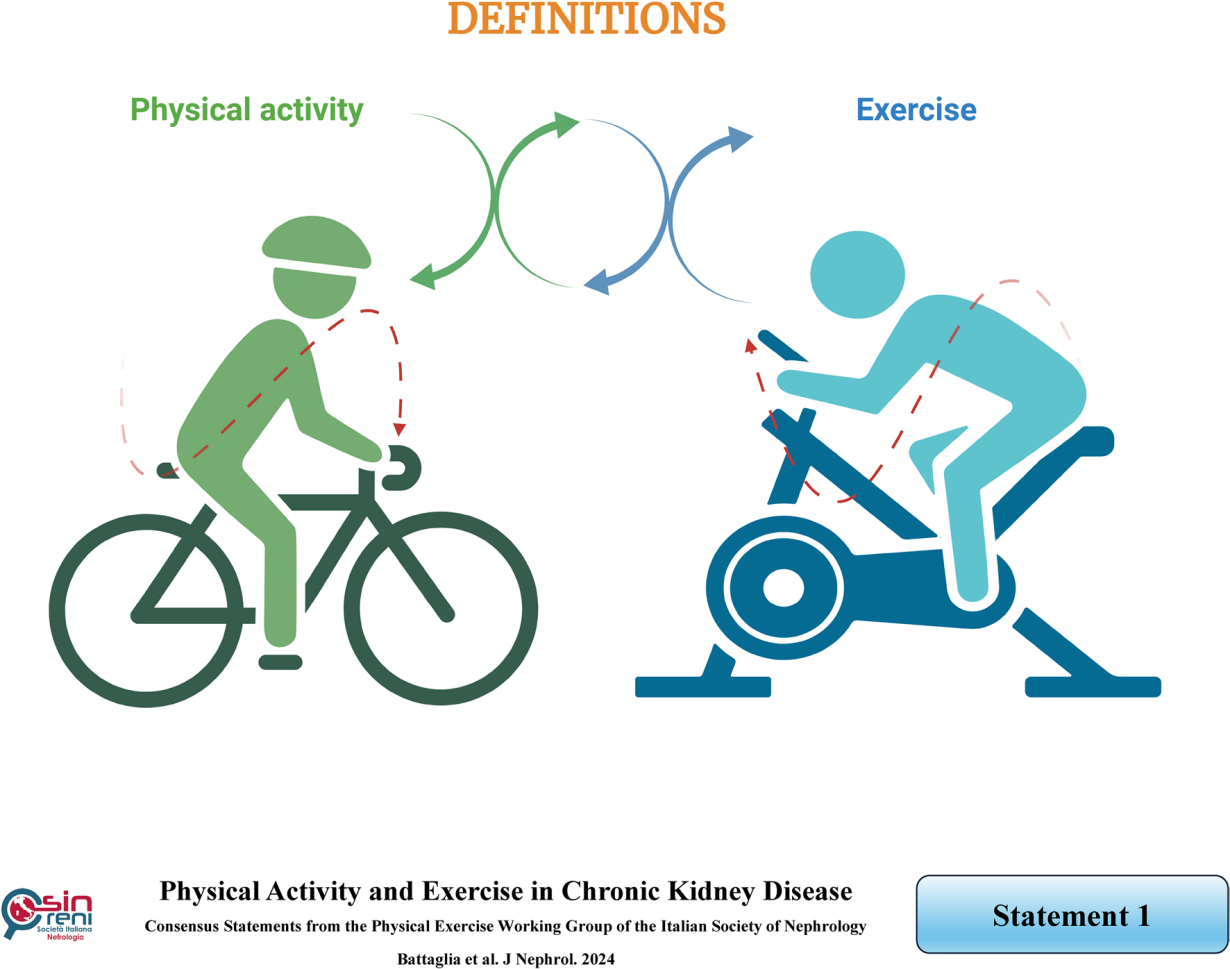


## Rationale

The term “physical activity” refers to any movement of the body produced by the contraction of skeletal muscles that increases energy expenditure compared to the baseline value (e.g., walking, climbing stairs). In contrast, “exercise” is a planned, structured, repetitive form of physical activity intended to improve or maintain physical fitness [5].

Before prescribing any form of exercise, it is essential to assess physical function, which is defined by the patient’s ability to perform routine daily activities [14]. Physical function differs from physical fitness, which refers to a set of attributes that people have or achieve related to their ability to perform physical activity, including cardiorespiratory fitness, muscle strength, and flexibility [11]. In detail, cardiorespiratory fitness is the capacity of the circulatory and respiratory systems to deliver oxygen during sustained physical activity. It is typically expressed as maximal oxygen uptake during exercise testing [5].

## Physical inactivity is prevalent in CKD patients and represents a modifiable risk factor for increased mortality and morbidity, as well as for reduced quality of life.


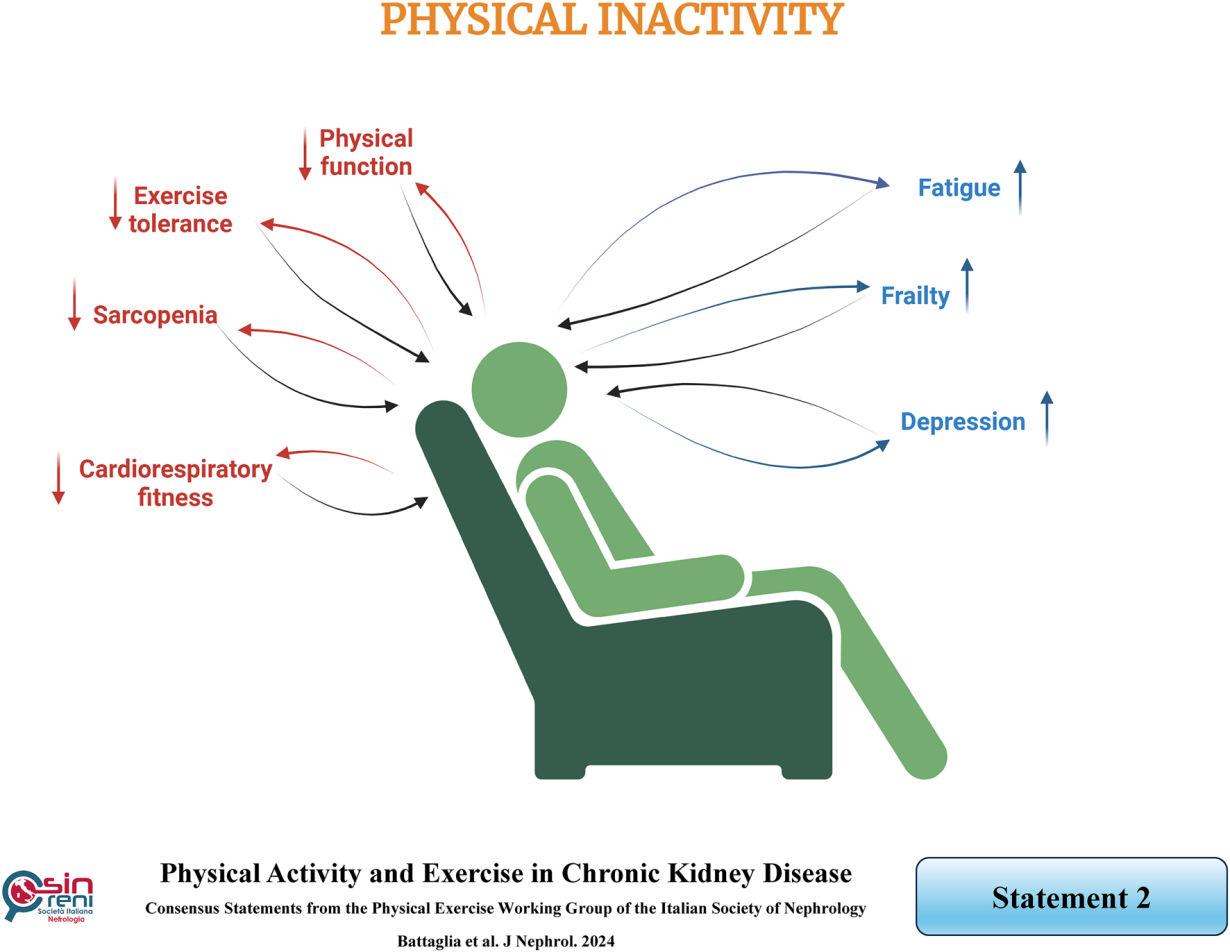


## Rationale

There is no specific definition of "*physical inactivity*" (also referred to as sedentarism). Some authors define this term as an insufficient level of physical activity compared to current recommendations, although there are often limited details available for CKD patients [11, 15].

Physical inactivity is regarded as the underlying network responsible for the presence of disease clusters, such as diabetes mellitus, cardiovascular disease, dementia, and depression, all of which share common pathogenetic mechanisms. This concept, known as the diseasome of physical inactivity, is also frequently observed in CKD patients [5]. Indeed, the risk of developing CKD is higher in inactive or minimally active individuals than in those engaging in moderate or high levels of physical activity [16].

On the other hand, when compared to the general population, CKD patients typically exhibit lower levels of physical activity, with some becoming completely inactive. Indeed, physical inactivity is present in all CKD stages and worsens as the disease progresses, reaching its peak in dialysis patients [17]. The leading causes contributing to the high prevalence of physical inactivity and sedentary lifestyle in CKD patients may be divided as follows [18]:

- Patient-related factors: physical (e.g., older age, female gender, higher number of comorbidities), psychological, cultural, and socio-economic;
- Disease-related factors: fatigue, depression, lack of energy, comorbidities, polypharmacy, uremia, chronic inflammation, insulin resistance, metabolic acidosis, anemia, Chronic Kidney Disease-Mineral and Bone Disorder (CKD-MBD), sarcopenia, protein-energy wasting and endothelial dysfunction [19];
- Treatment-related factors: CKD therapies or dialysis schedule, health staff attitude, lack of physical activity assessment and a low rate of exercise counseling, exercise-associated untoward outcomes, availability of tutoring, suitable environments, and equipment.

In turn, physical inactivity leads to reduced physical function, exercise tolerance, muscle mass and strength, and cardiorespiratory fitness while increasing fatigue, frailty, and risk of depression [20, 21]. These factors create a vicious circle of reduced physical activity, which can be broken only by exercise implementation.

## Physical activity and exercise significantly benefit CKD patients, particularly in improving physical function, cardiorespiratory capacity, muscle strength and overall quality of life


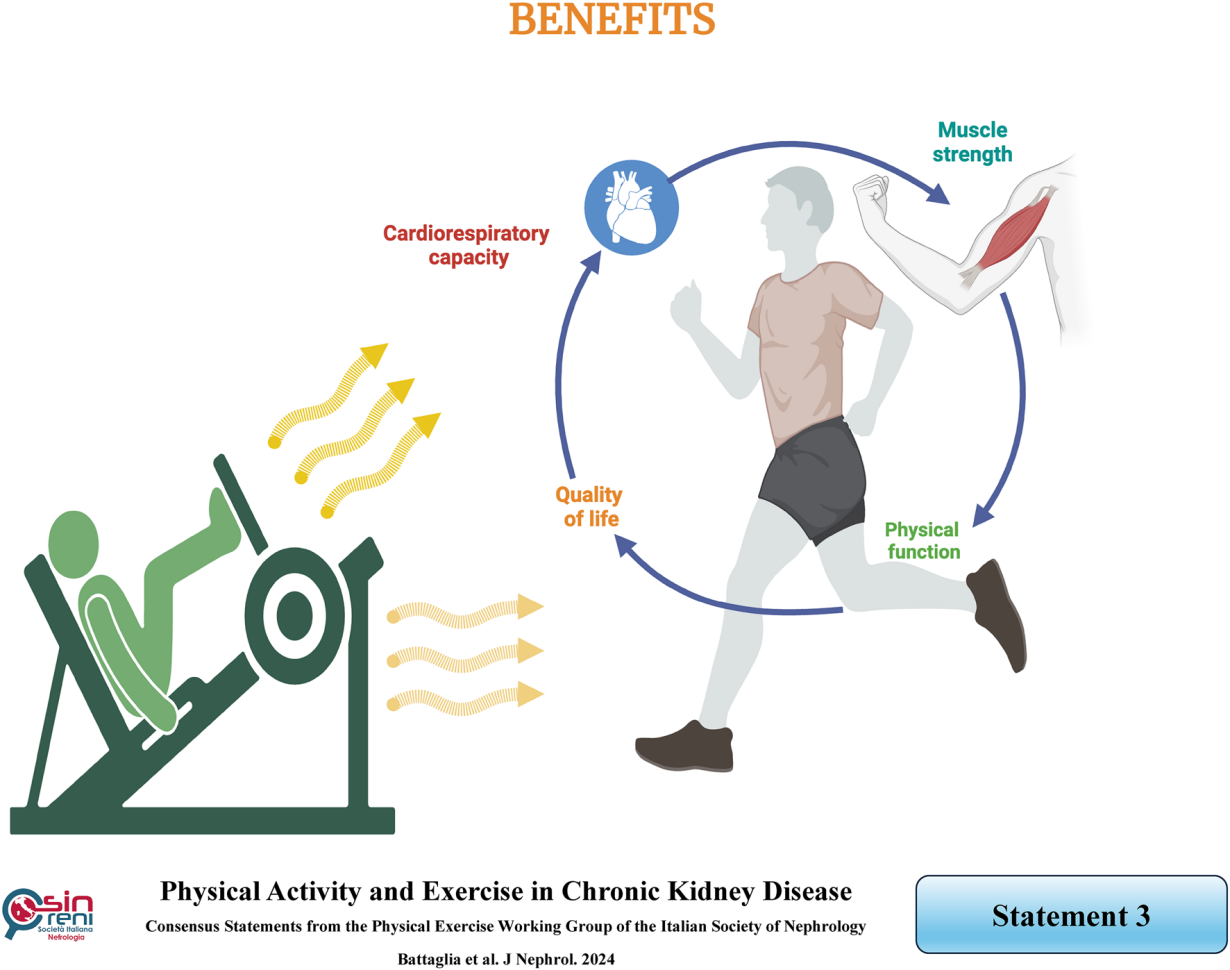


### Rationale

Regular physical activity is associated with reduced mortality and morbidity in the general population. Physical activity plays a crucial role in primary prevention in the general population and secondary and tertiary prevention for specific categories of patients. These considerations can also be applied to CKD patients. Indeed, a growing body of literature suggests that physical activity and exercise offer numerous benefits to CKD patients [5].

Firstly, randomized controlled trials have demonstrated improvements in physical function, cardiorespiratory fitness, muscle strength, and quality of life [22–25]. Secondly, observational studies have described reduced mortality risk and a nephroprotective effect. The mechanisms underlying a possible nephroprotective effect are diverse and could be related to controlling blood pressure, body weight, and glucose tolerance [21, 26, 27]. Thirdly, some experimental studies suggest the existence of a muscle-kidney cross talk, where muscle contraction stimulates the release of factors that promote cell growth within the damaged kidney [28]. Finally, physical activity enables the management of the several comorbidities that characterize CKD patients by improving cardiometabolic, neuromuscular, and cognitive function [27]. However, it is worth noting that further long-term, randomized controlled studies are needed to precisely define the effects of exercise on relevant outcomes such as mortality and CKD progression.

## Physical exercise is safe in CKD patients when appropriately performed


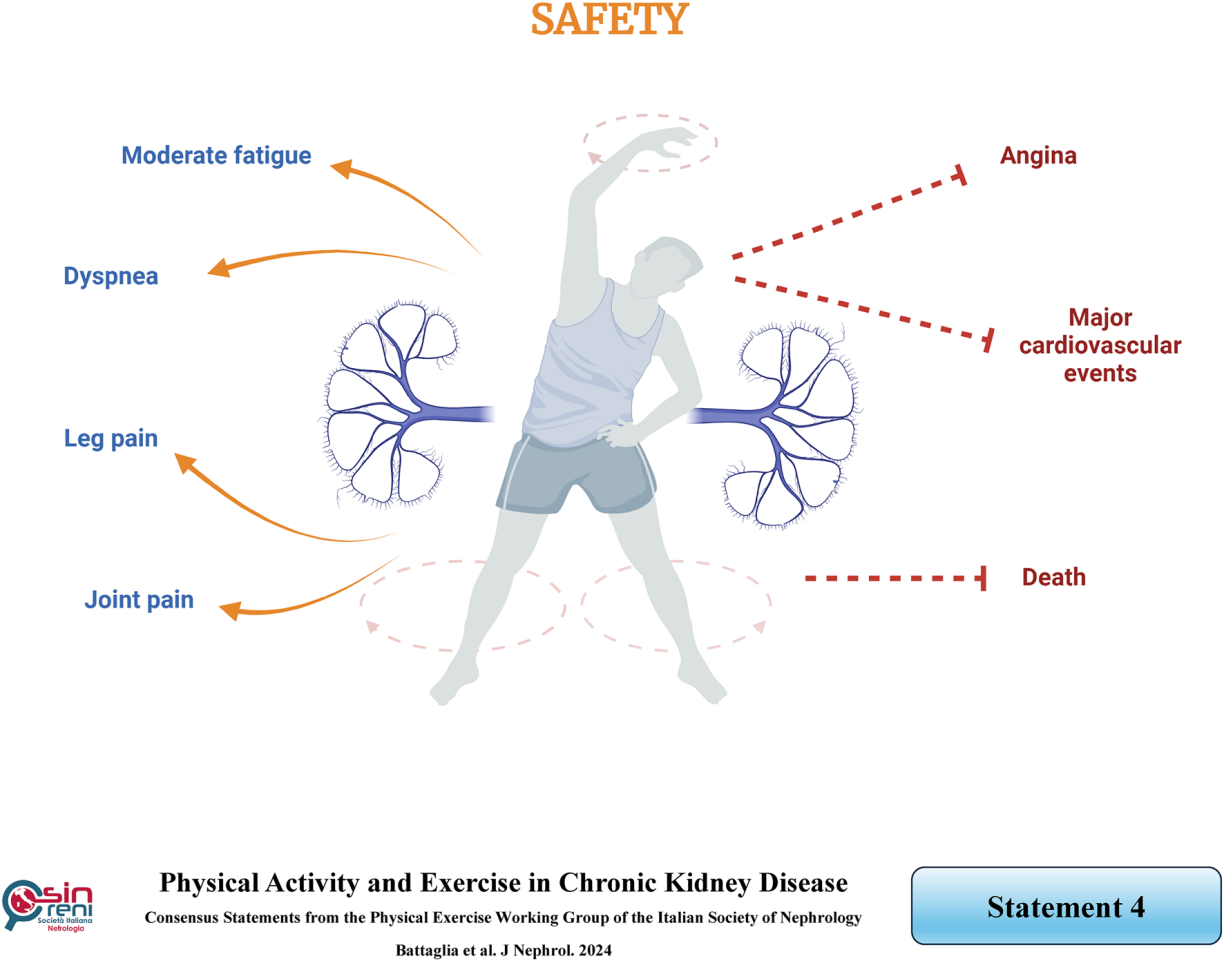


### Rationale

Cardiovascular disease is the leading cause of death in patients with CKD. Notably, for CKD patients on dialysis or conservative therapy and KTRs, the predominant concern is a cardiovascular event such as arrhythmia, heart attack, and hypertension. Remarkably, no cardiac events have been reported in any published exercise training studies involving hemodialysis patients [11]. However, a limited number of hypotensive episodes may occur in patients engaging in intradialytic exercise, likely attributed to the attenuation of the cardiovascular response to sympathetic nervous system activation [29]. These episodes are more frequent during high-intensity than moderate-intensity exercises [11]. Lin et al. demonstrated in their randomized controlled trial (RCT) that moderate-intensity intradialytic exercise for 12 weeks did not cause adverse events, not even hypotension. The reason likely lies in the acute physiological response to exercise, which helps increase circulating volume and improve hemodynamics by promoting greater reuptake of blood from tissues [30]. Similarly, in the EXCITE study, during low-intensity home-based exercise, moderate-intensity symptoms such as moderate fatigue, leg pain, dyspnea, and joint pain were recorded in 54% of sessions performed by 38 patients, while angina or significant symptoms were not reported [31].

When it comes to patients on peritoneal dialysis engaging in exercise training with both a full or empty abdomen, a few cases of leaks, hernias, or catheter dysfunction, but no severe adverse events, have been reported. Increasing abdominal musculature through exercise likely contributes to increased resistance against hernias and leaks [32].

Although less studied, exercise training can be deemed safe for patients with stage 3–4 CKD [33]. Indirectly, sedentary patients face a higher risk of cardiovascular events than those who exercise during physical inactivity or activity periods [11].

Currently, there is limited literature on the safety of exercise for KTRs. Studies with longer follow-ups and larger sample sizes are needed to comprehensively understand the effects of exercise on cardiovascular events, death, and risk of transplant rejection [34]. However, based on the clinical experience, no significant adverse events correlating with regular physical exercise have been identified in KTRs so far [8, 35].

Nevertheless, the current literature does not allow for definitive conclusions on whether CKD patients are at higher risk of exercise-induced death than the general population [14]. Therefore, as a precaution, before starting exercise training, CKD patients should undergo medical evaluation by a sports medicine physician [11] to assess multiple parameters, including exercise suitability, medical history, current therapy, physical evaluation, electrocardiogram, and laboratory tests.

## Physical function assessment can be performed routinely through physical performance tests and patient-focused questionnaires


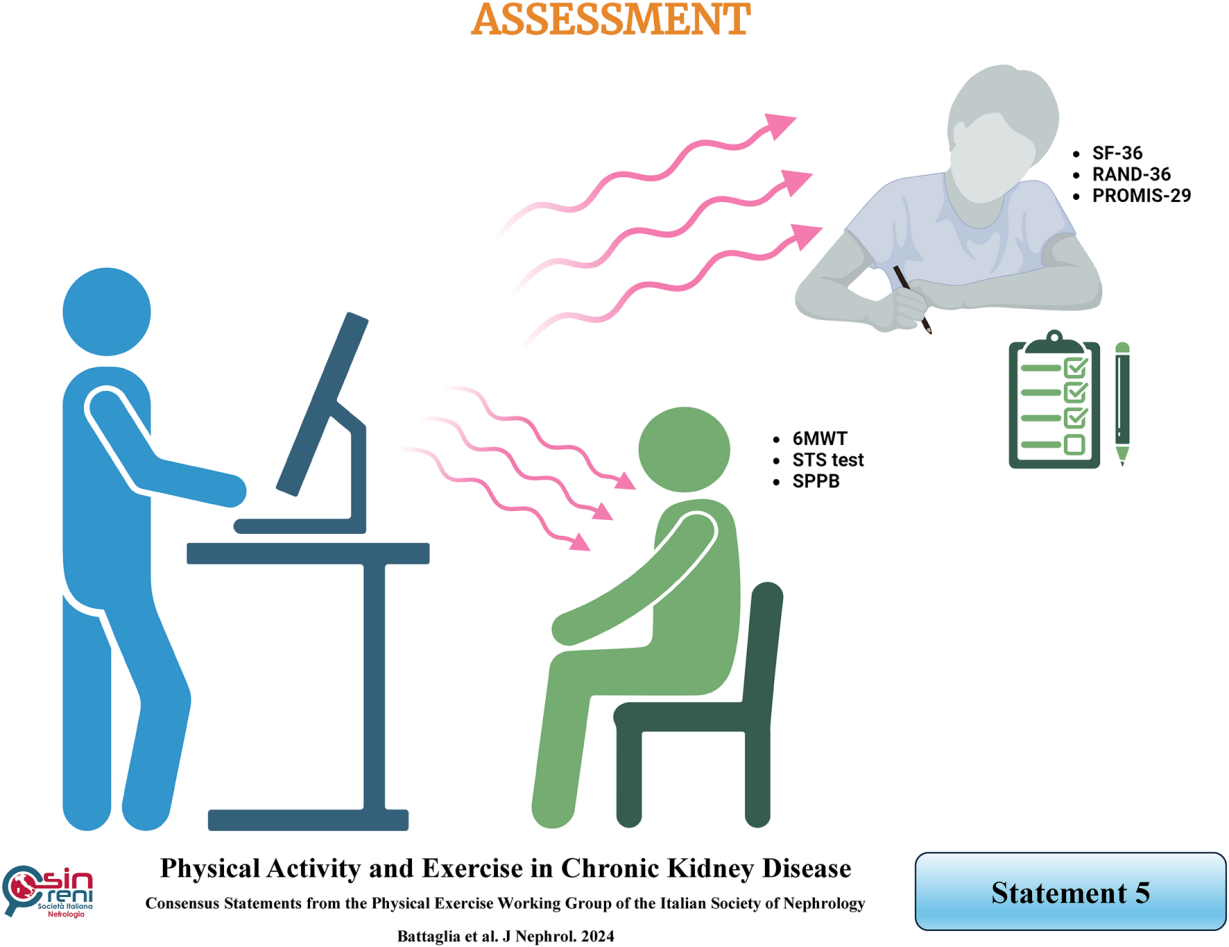


### Rationale

Physical function is typically assessed through objective physical performance tests or self-reported physical function questionnaires, both of which are significantly associated with survival in CKD patients [36]. It should be evaluated at least annually, regardless of physical activity levels, to obtain a more comprehensive health status assessment of CKD patients, especially in end-stage kidney disease and dialysis patients in whom physical function declines more rapidly [37]. Furthermore, physical function should be assessed before prescribing any physical activity or exercise program (see statement 10) to tailor the prescription, and during the interventions, every three or six months, to monitor improvement.

*Physical performance tests,* such as the Six-Minute Walk Test (6MWT), the Sit-To-Stand (STS) test, and the Short Physical Performance Battery (SPPB), should be included in the routine management of CKD patients on conservative or dialysis treatment and KTRs (Table 1). The 6MWT and STS tests have often been used to assess aerobic capacity and lower limb function [38] in multiple recent clinical trials conducted primarily in patients on dialysis [39–41] Interestingly, in CKD patients, a strict correlation was found between each reduction of 1 ml/min/1.73 m^2^ in eGFR and a 1.5 times increase in the odds that patients would be unable to rise from a chair [42]. Meanwhile, the more comprehensive SPPB can provide measurements not only of the strength and gait domains but also of the balance domain [43, 44]. This test has been reported to be a valuable tool for predicting falls, hospitalization, and mortality in CKD and dialysis patients and KTRs [45–50]. In a CKD cohort, poor physical performance, as assessed by low SPPB scores, was found to be statistically associated with higher severity of renal dysfunction [51].

**Table 1:** Characteristics of tests for assessing physical function in CKD patients

Domains	Test	Description
Aerobic Capacity	*SIX MINUTES WALKING TEST (6MWT)*	– It involves measuring the distance an individual can cover over six minutes on a flat, rigid surface without interruption – The marked path is often calculated using cones and can be as simple as a corridor in a ward or dialysis unit for convenience – The participant should walk self-paced and rest in case of any difficulties while traversing back and forth along the designed path
Lower Limb Function	*SIT TO STAND TEST (STS)*	– It assesses the number of completed cycles in which an individual from a sitting position stands up and then sits back down over 30 s – A straight-back or folding chair without armrests (seat 44 cm high), commonly utilized at hospitals, and a stopwatch (app available on any mobile phone) to check the time are necessary to perform such tests – During the procedure, the individual sits in the middle of the chair with his/her feet shoulder-width apart and flat on the floor. The arms are crossed at the wrists and held close to the chest
Strength, Gait, and Balance	*SHORT PHYSICAL PERFORMANCE BATTERY (SPPB)*	– It consists of three sequential, functional tests: gait speed, chair stand and balance tests 1) Gait speed test measures the time it takes for an individual to walk four meters at the usual pace; 2) Chair stand test assesses the time required to perform five consecutive repetitions of standing up and sitting down in a chair as quickly as possible without using the arms; 3) Balance test evaluates an individual's ability to stand upright in three different positions over 10 s: feet together, with one foot partially forward, and one foot forward – Each test score ranges from 0 (inability to perform the task) to 4 points (best test performance). By summing up the three scores, the SPPB total is calculated and scored from 0 (worst performance) to 12 points (best performance) – According to SPPB scoring, a patient's performance is categorized in: a) Three classes: scores 0–6 (poor performance), scores 7–9 (moderate performance), and scores 10–12 (good performance); b) Four classes: scores 0–3 (disability/very poor performance), scores 4–6 (poor performance), scores 7–9 (moderate performance), and 10–12 points (good performance).

Taken together, these findings suggest that SPPB can be considered a reliable measurement instrument of functional capacity, lower extremity strength, and balance in the CKD population, consistent with results from other settings.

Self-reported physical function questionnaires primarily encompass the 36-item Short Form Health Survey (SF-36) [52], RAND-36 [53] or Patient-Reported Outcomes Measurement Information System (PROMIS) Global Health and PROMIS-29 [54]. It is advisable to use the adapted versions of SF-36, such as the 36-item Kidney Disease Quality of Life Survey (KDQOL-36) for CKD patients or its Short Form (KDQOL-36-SF) for dialysis and CKD patients [55]. These two modified subjective tests include specific items tailored to patients with CKD [56]. However, numerous self-report tools are available to capture patient-focused information on activities of daily living, health-related quality of life, kidney disease quality of life, and the sickness impact profile [57–59].

## Monitoring physical activity levels using self-report instruments or objective measures is advisable to counteract sedentary behavior


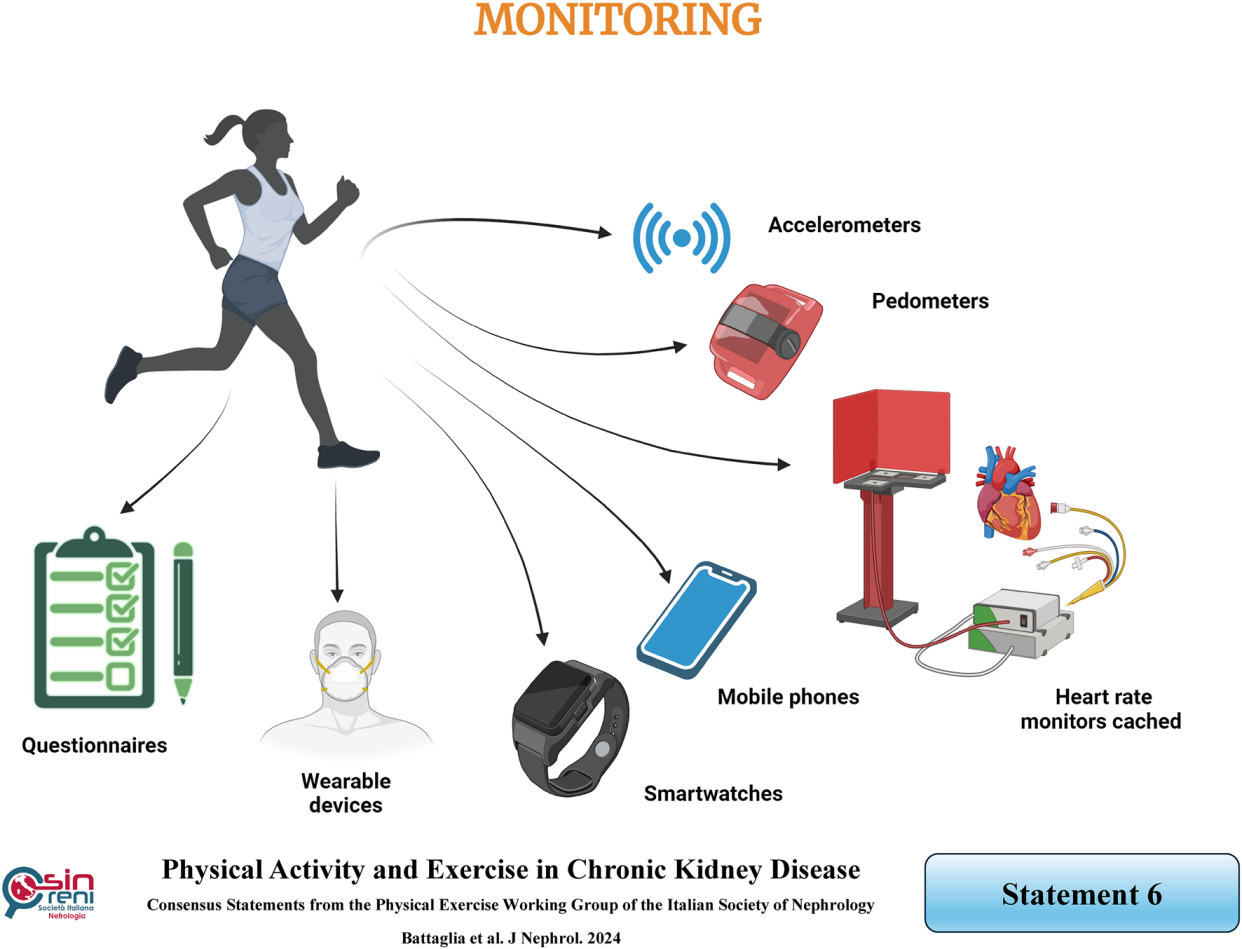


### Rationale

While some physical activity is undoubtedly better than none, it is crucial to accurately identify the patient’s physical activity levels or sedentary behavior using validated activity questionnaires (Table 2). Logs or diaries collect hour-by-hour or activity-by-activity information [60].

**Table 2:** Features of self-report instruments assessing physical activity in CKD patients

Tool	Characteristics
Global Physical Activity Questionnaire (GPAQ)	– It is a short self-report questionnaire that explores physical activity, occupation, and walking pace domains for 11 items – It is scored in four categories: "active", "moderately active", "moderately inactive", or "inactive"
Physical Activity Vital Signs (PAVS)	– It is a formally validated screening tool endorsed by the American College of Sports Medicine and recommended for use in CKD patients and KTRs as an audit measure by the UK Kidney Research Consortium Clinical Study Group for exercise and lifestyle – Two open questions are included: 1) "On average, how many days per week do you engage in moderate to strenuous exercise like a brisk walk?"; 2) "On average, how many minutes do you engage in exercise at this level?"
94-item Human Activity Profile (HAP)	– It includes a list of activities requiring from 1 to 10 METs, rated in three options: (a) "*Still doing this activity*"; (b) "*Have stopped doing this activity*"; (c) "*Never did this activity" *” – Three indexes are derived: *1)* Total Activity Score (TAS) is the sum of activities (option a + b), ranging from 0 to 94 points. It is scored in three categories: "inactive" (≤ 53 points), "moderately active" (54–73 points), and "active" (≥ 74 points) *2)* Maximum Activity Score (MAS) is the sum of the more intense activities (option a); *3)* Adjusted Activity Score (AAS) is calculated by subtracting from the MAS the responses "Have stopped doing" (option a − b)

*CKD* chronic kidney disease, *KTRs* kidney transplant recipients, *MET* metabolic equivalent of task

Global Physical Activity Questionnaire (GPAQ) [61], a NICE-recommended survey is instrumental in identifying those who are inactive and in need of support. In a validation study for CKD patients, compared to accelerometry, GPAQ demonstrated a sensitivity, specificity, and accuracy of 54.6%, 96.6%, and 85.0%, respectively [62].

Other valuable questionnaires, such as Physical Activity Vital Signs (PAVS) [63] and 94-item Human Activity Profile (HAP) [64] can be used to gauge the level of physical activity. Specifically, HAP is a comprehensive questionnaire that evaluates activities across various energy requirements and has been validated in CKD patients. Johansen et al. demonstrated, for the first time, a strong correlation between HAP and seven-day accelerometry, the gold standard measure of physical activity (*r* = 0.78, *P* < 0.0001) in dialysis patients [65].

From the HAP assessment, both Maximum Activity Score (MAS) and Adjusted Activity Score (AAS) may be derived [66], with the latter as a measure of the individual's habitual physical activity level. These scores should be compared with established normative data based on age and gender. In dialysis patients, lower maximum activity score and adjusted activity score were found to be correlated with a higher risk of death [67]. Surprisingly, habitual physical activity, assessed by adjusted activity score, appears to be a stronger predictor of quality of life in dialysis compared to healthy subjects [68].

However, HAP is time-consuming, with over 94 items to address, thus limiting its widespread use in clinical practice. Therefore, a shorter, adapted version of the HAP questionnaire has been proposed [69] and successfully tested in CKD, dialysis, and peritoneal patients, as well as in KTRs, showing different physical activity levels across the CKD population.

Physical activity should be monitored daily, but patient compliance in collecting questionnaire data tends to decrease over time, even with the briefest and most easily administered questionnaire. To overcome this limitation, technological advancements and miniaturization have led to increased ambulatory movement registration techniques in recent years. Daily physical activity can be continually measured using accelerometers, pedometers, or heart rate monitors cached in wearable devices, smartwatches, or mobile phones [70, 71]. These technologies allow long-term monitoring and provide a positive stimulus to increase physical activity levels.

## Protein restriction does not blunt or prevent the favorable effects of exercise on muscle strength and mass in CKD patients


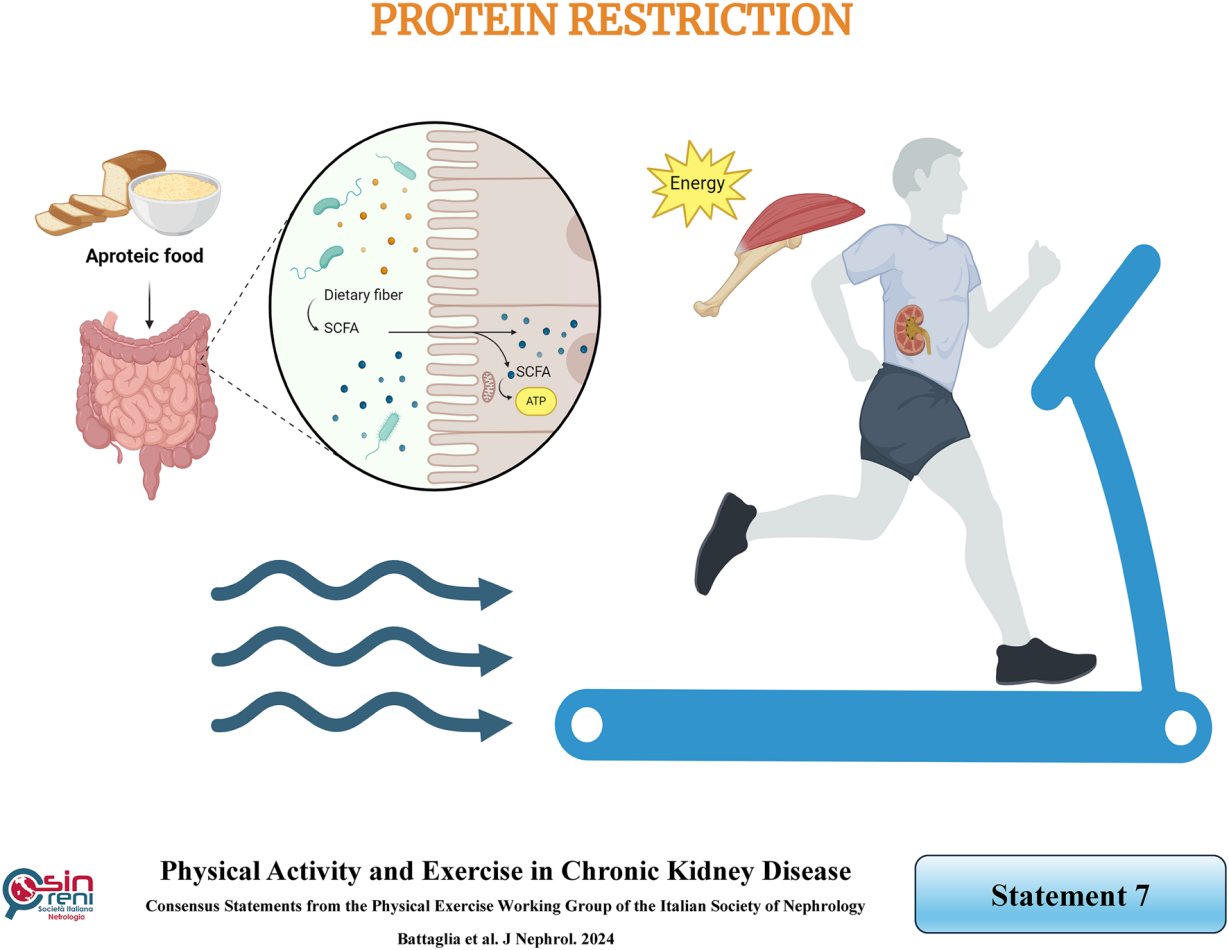


### Rationale

A low-protein diet (LPD) plays a pivotal role in CKD patients' conservative management, and if energy intake is adequate, it does not affect muscle mass. Castaneda et al. conducted an RCT in which patients with moderate CKD on an LPD (0.6 g/kg of body weight per day) were randomized to resistance training (*n* = 14) or no exercise for 12 weeks. Total body potassium and type I and II muscle-fiber cross-sectional areas increased in patients on an LPD who performed resistance training compared with those who were not. Accordingly, leucine oxidation, serum pre-albumin levels, and muscle strength increased. These data show that the improvement of muscle mass and strength and nutritional status induced by resistance training occurs even during protein restriction regimens [72]. A secondary analysis of the above mentioned study investigated mitochondrial biogenesis by quantitative real-time PCR of mitochondrial DNA copy number in the vastus lateralis muscle. A marked increase in muscle mitochondrial DNA was observed in the exercise group, and they were positively correlated with the changes in fiber cross-sectional area of types I and II muscle fibers [73].

These findings suggest resistance training enhanced mitochondrial content in patients with moderate-to-severe CKD on a low-protein diet. In summary, a low-protein diet does not represent a condition that limits the favorable effects of exercise training on muscle ultrastructural, metabolic, and morpho-functional features. Moreover, although a low protein diet “per se*’*” may reduce protein synthesis, it induces favorable effects on metabolic acidosis and insulin resistance associated with CKD, preventing their pro-catabolic effects on protein metabolism [74].

## Dietary supplements combined with exercise are an effective strategy for preventing sarcopenia or protein-energy wasting in CKD patients, including those on dialysis


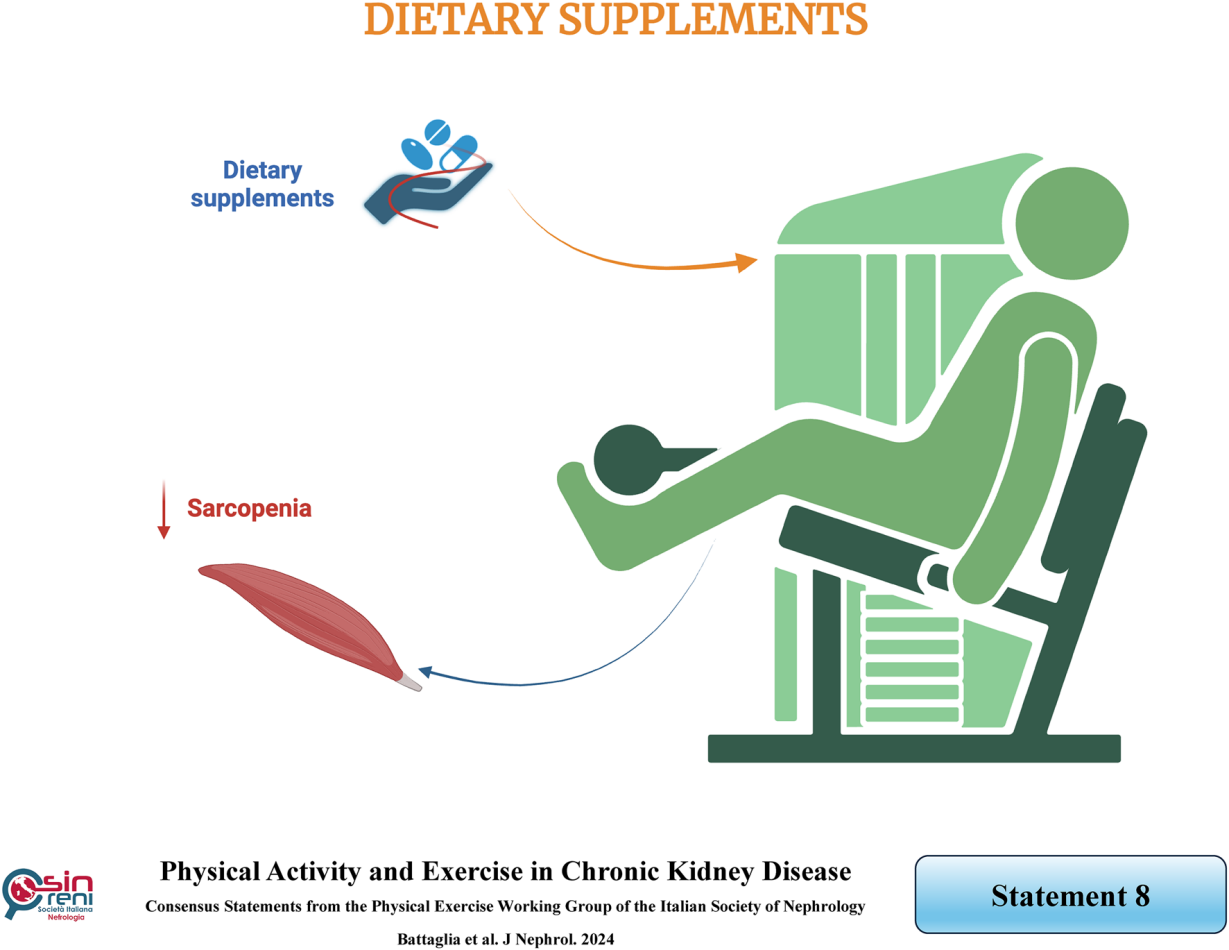


### Rationale

Evidence shows that the loss of skeletal muscle mass in dialysis patients may be due to changes in intracellular signaling that involves the insulin receptor substrate/phosphatidylinositol 3-kinase/Akt pathway, which leads to reduced mammalian target of rapamycin (mTOR) stimulation and consequently decreased protein synthesis. Exercise can reverse these changes and increase protein intake. The muscle protein synthetic rate in response to protein feeding is impaired in hemodialysis patients when studied on non-dialysis days [75]. This kind of anabolic resistance to dietary protein is well known also in the elderly. Conversely, physical exercise represents the physiological anabolic stimulus for skeletal muscle, while inactivity induces muscle mass and strength loss.

Therefore, it is conceivable that coupling adequate nutrition with physical exercise can prevent muscle loss in dialysis patients more effectively than nutritional intervention or physical exercise applied separately. While no effect on lean body mass was found when exercise training alone or nutritional supplementation alone was implemented, a significant improvement in body weight and muscle strength was observed when resistance exercise was coupled with intradialytic oral supplementation of protein and energy [76].

Protein kinetics in dialysis patients following oral nutritional supplementation and resistance exercise were investigated using stable isotope. The results showed that exercise significantly increased the protein anabolic effects of oral intradialytic nutritional supplementation [77]. Moreover, an increased rate of protein synthesis was reported in untrained subjects when resistance exercise was coupled with whey protein intake. This intervention induced a higher level of phosphorylation of mRNA translational signaling proteins, namely the mTOR pathway, than that related to exercise alone [78]. Activation of mTOR signaling by whey protein intake after resistance exercise occurs in a dose-dependent manner [79]. Protein intake and exercise training during the hemodialysis session do not have a negative impact on dialysis efficacy nor do they compromise the removal of uremic toxins [80].

In summary, protein supplementation and resistance exercise training can likely stimulate protein synthesis by activating pAkt and mTOR pathways. In contrast, aerobic exercise training and energy supplementation have favorable effects on mitochondrial and energy metabolism, reducing protein degradation. Coupling protein-energy supplementation with exercise training may contribute to maintaining muscle mass and counteract sarcopenia [6]. This is one more example of the usefulness of a team that includes a dietitian/nutritionist and exercise professional/physiotherapist to implement as much as possible proper nutrition and tailored physical activity, both within dialysis centers or hospital facilities and in a home-based setting.

## Barriers to physical activity in the CKD population are well known: each nephrology and dialysis unit needs to identify its center-specific barriers and establish a detailed plan for overcoming them


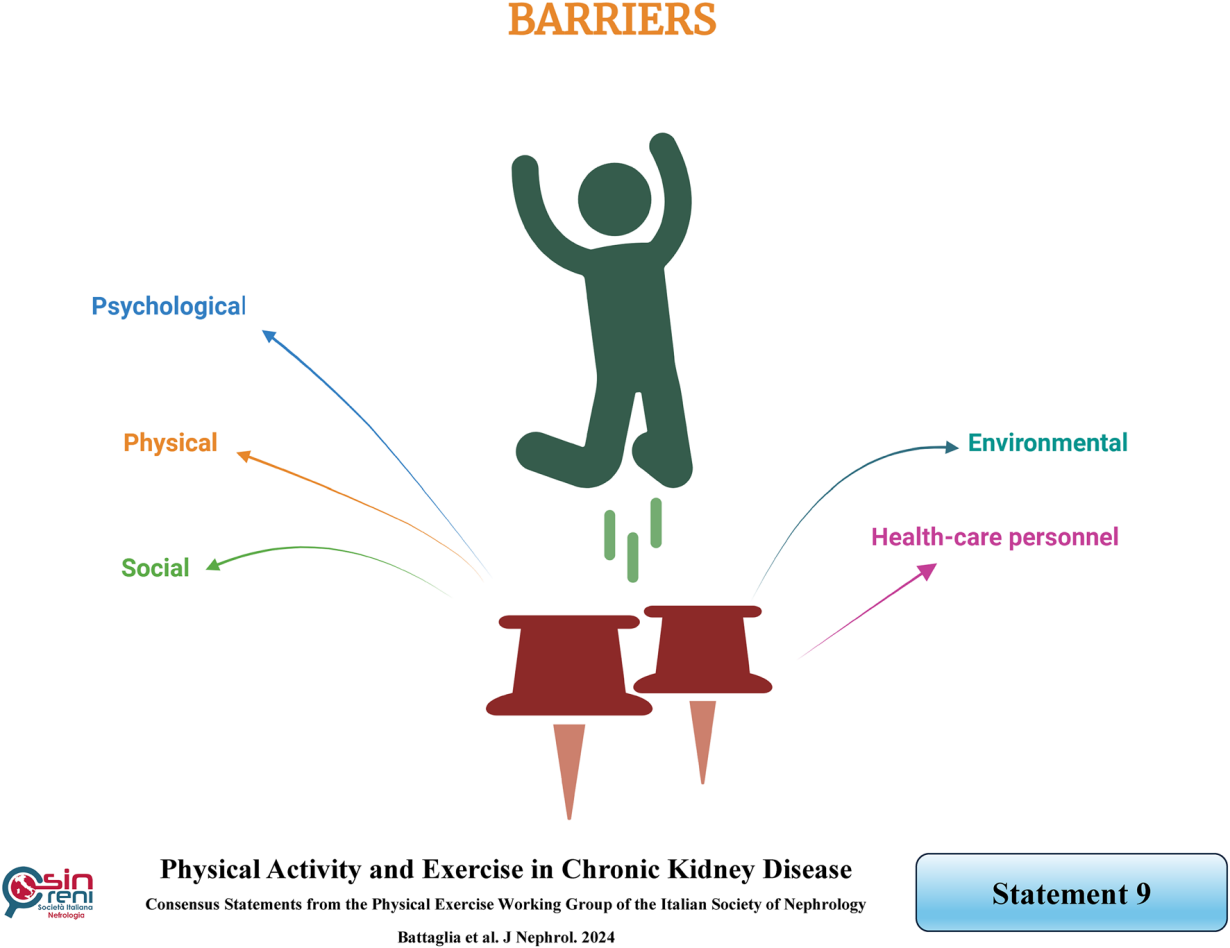


### Rationale

In the last decade, a sufficiently broad body of literature has characterized and defined the barriers that limit the implementation of physical activity in CKD patients and has outlined appropriate strategies to overcome them (Table 3). Generally, barriers to physical activity are quite common, with the vast majority of patients reporting at least one barrier and frequently two or more. Substantial numbers of patients endorse many barriers. Both a more significant number of reported barriers and the endorsement of several specific barriers were associated with lower levels of physical activity [81].

**Table 3:** Barriers to physical exercise

Barriers	Descriptions
Psychological	Negative feelings (e.g., helplessness, sadness, demoralization) Feeling too old Fear of getting hurt Lack of motivation
Physical	Fatigue (for HD patients: on dialysis or non-dialysis days) Shortness of breath Pain (e.g., chest pain) Comorbidity conditions (e.g., heart failure, stroke attack, ulcers on legs and feet, amputation)
Social	Lack of exercise partner Family concerns Lack of time Inability to travel Too many medical appointments
Environmental	Weather Cost No safe place for exercise
Health-care personal	Lack of counseling Negative attitude Lack of time to discuss with patients Lack of interest

A simplified and holistic approach to overcoming these barriers has recently been proposed [82].

Each nephrology center must evaluate its population through a four-step program:

1. Evaluate the level of physical activity (see statement 6)
2. Identify the barriers related to patients;
3. Identify the barriers related to the health care staff;
4. Plan an appropriate strategy for overcoming the barriers.

For assessing the presence of *patient-related barriers,* the questionnaire by Delgado & Johansen [83] can be helpful. It includes questions related to various categories of disease- and patient-specific barriers to physical activity, such as psychological barriers, physical barriers, economic barriers, lack of time, and comorbidities. Short and validated screening tools for assessing depressed mood, recent falls, and functional impairment are readily available, and they can be easily incorporated into clinical care management.

The *staff’s attitude* toward physical activity counseling may be analyzed using the Fiaccadori questionnaire [84], a modified version of Johansen's. It comprises 17 questions, 13 of which are applicable to nurses, focusing on the opinions and practices of dialysis personnel regarding physical activity counseling.

It is noteworthy to consider that the different barriers may influence each other. Patients who perceive the staff as supportive are likelier to have a positive attitude towards physical activity. Conversely, it has been demonstrated that patients in poor health, endorsing multiple barriers to physical activity, may benefit less from a proactive staff attitude compared to patients in better general conditions with fewer barriers [85]. Moreover, a positive interaction between a proactive attitude of healthcare providers and the clinical setting (e.g., availability of trained exercise program supervisors) has been reported to stimulate patients' willingness to increase physical activity [85].

In order to *plan an appropriate strategy*, the stakeholders, including patients, dialysis staff, and nephrologists, must be involved [8].

The first step is to address a critical gap in the patient's knowledge of the health benefits of physical activity, which becomes a primary target for intervention. Patients are often unaware of the recommended frequency and duration of physical activity [87]. On the other hand, although health professionals are frequently aware of the benefits of physical activity, they may need a more proactive attitude [7]. There is a passive attitude among healthcare providers towards inquiring about patients' physical activity levels. Indeed, although nephrologists generally recognize the importance of increasing physical activity, they usually do not actively encourage it [88]. This reluctance is often attributed not only to a lack of time but also an inability to counsel [34, 89]. Therefore, to enhance physician confidence in counseling, guidelines should explicitly recommend which methods to adopt [66, 90]. Additionally, better use of available materials could assist nephrologists in prescribing physical activities such as those designed for patients on dialysis therapy.

A second step involves carefully evaluating this specific population and applying for an individualized program with prior testing of an individual's capabilities (see statement 10).

## It is crucial to personalize physical activity prescriptions, which can be provided by a nephrologist, while in the presence of functional limitations, a physiotherapist or sports medicine physician consultation is generally needed


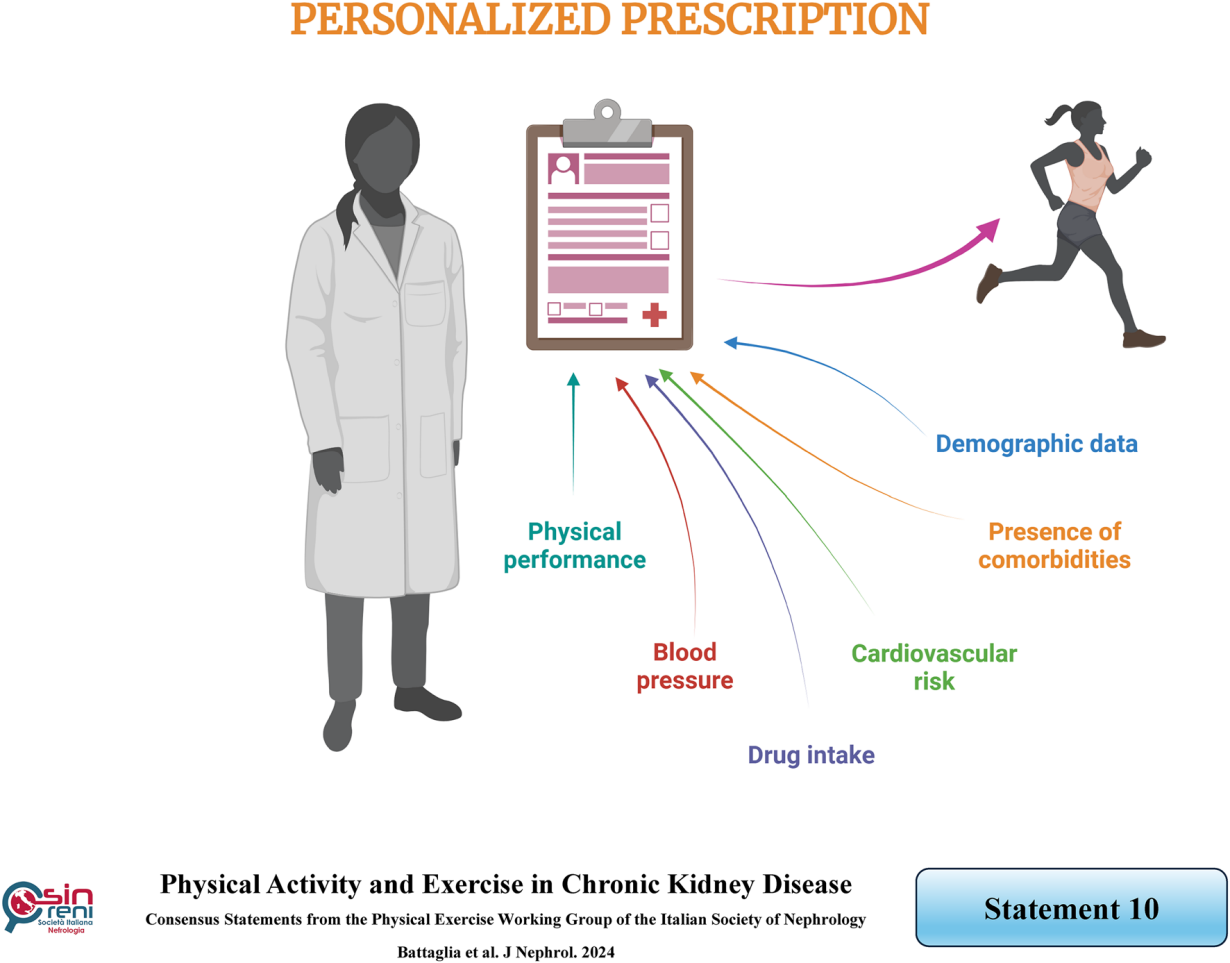


### Rationale

Physical activity may be prescribed in CKD patients, considering primarily their general clinical conditions and current physical performance. Firstly, information concerning demographics, the presence of comorbidities, and drug intake should be collected before providing a personalized prescription for physical activity. This approach helps to avoid generic advice that may lack efficacy and ensures that physical activity is tailored to the individual's needs and circumstances. For instance, patients with high comorbidity need to be encouraged to increase overall physical activity by boosting daily step counts, moving between rooms, and standing longer. Additionally, dialysis schedules and medications should be re-evaluated to achieve better control of factors such as fatigue, pain, and sadness.

However, it is crucial to exclude patients with high or acute cardiovascular risk from participating in exercise training, specifically those with recent myocardial infarction, unstable angina, uncontrolled arrhythmia, symptomatic severe aortic stenosis, suspected aortic dissection, and poorly controlled severe hypertension [91]. As per the American College of Sports Medicine, exercise is discouraged if systolic blood pressure is greater than or equal to 200 mmHg and diastolic blood pressure is greater than or equal to 110 mmHg [11].

Regarding physical performance, as previously reported, various tests can identify this level, among which SPPB provides objective measurements comparable with other populations. If the SPPB score is moderate or high, to enhance patients' compliance, the needs and requests of patients regarding the type of physical activity should also be considered. Indeed, no differences in mortality, hospitalization, or disease progression are observed among different types of physical activities for CKD patients without absolute contraindications [8].

However, to achieve the desired goal, similar to the dosage of a drug, a tailored prescription is essential for CKD patients with physical limitations [92]. In-depth rehabilitation treatments, supervised by physiatrists and/or physiotherapists and/or sports medicine physicians and/or kinesiologists, are crucial to improving the physical function of frail, comorbid elderly individuals, including those with CKD [93].

## Exercise training for CKD patients can be undertaken alone or in groups, at home, in a dialysis unit, or in a sports facility


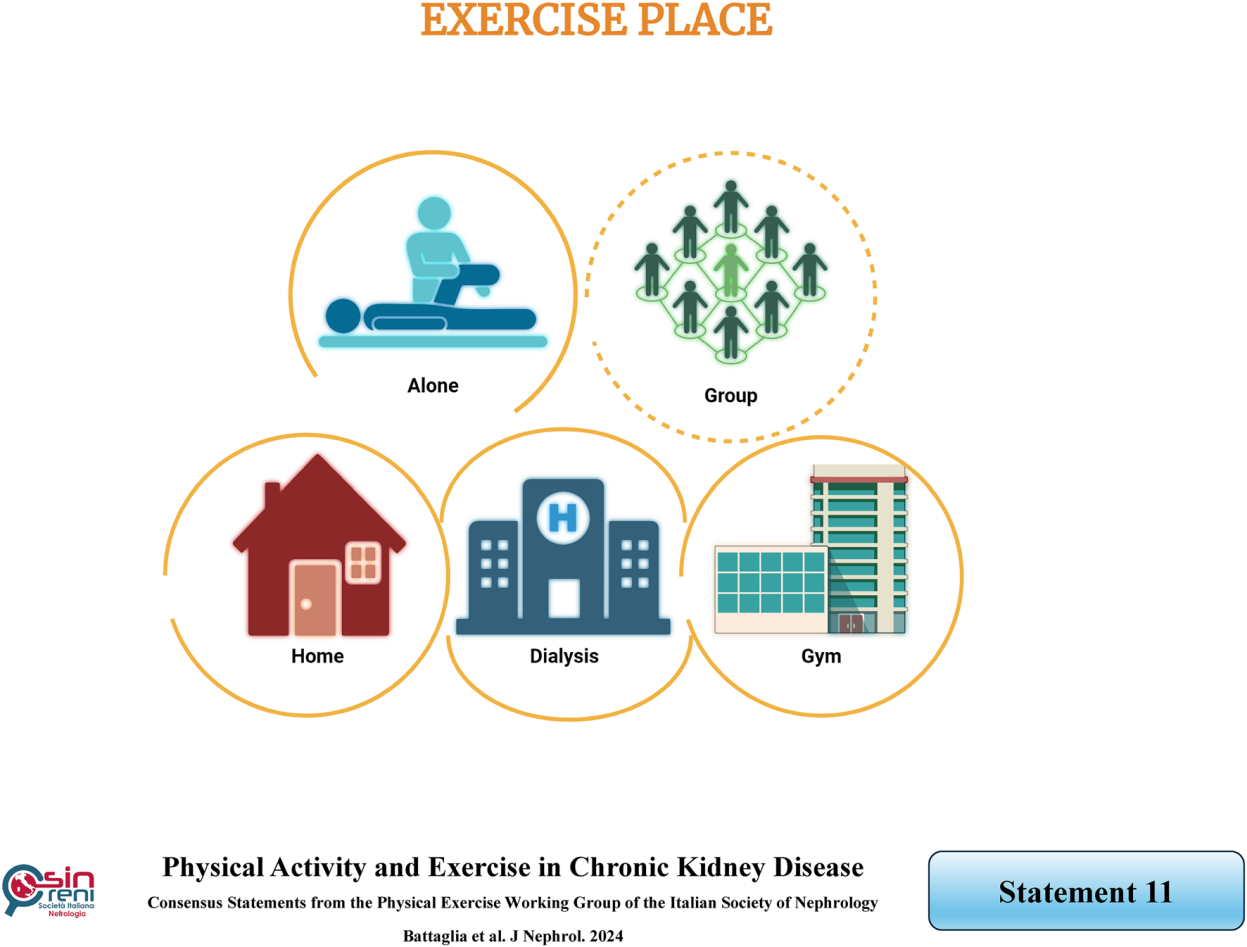


### Rationale

Over the years, several studies have explored various methods to improve health status and quality of life with exercise training in CKD patients on conservative or dialysis treatment and in KTRs. These studies evaluate the optimal location for activity (i.e., home, dialysis, or sports facility) (Table 4).

**Table 4:** Exercise training in CKD settings

Characteristic	Example	Population
*Location*		
Facility or Home	All types of exercise (aerobic, resistance, and flexibility)	CKD patients KTRs HD patients PD patients
Intradialytic or immediate pre/post-dialysis session	Cycle ergometer TheraBand (elastic bands) Free-weight Dumbbells Weighted ankle cuff	HD patients
*Type*		
Cardiovascular or endurance (Aerobic)	Cycling Walking Running Swimming	CKD patients KTRs HD patients PD patients
Strengthening or Resistance	Weight machines Free weights Bodyweight exercise	
Stretching or Flexibility	Static stretching Dynamic stretching Breathing exercise	

*CKD* chronic kidney disease, *KTRs* kidney transplant recipients, *HD* hemodialysis, *PD* peritoneal dialysis

Exercise can be performed either in the facility or at home for patients with CKD on conservative therapy and patients on peritoneal dialysis [11, 21]. The same applications are valid for hemodialysis patients, who can exercise on dialysis days (intradialytic or immediately pre/post-dialysis session) or interdialytic days, whether in the dialysis facility or home-based [94, 95].

*Intradialytic exercise* offers several advantages. It does not disrupt usual care or require additional dialysis time, as no patient wants to spend more time in the dialysis unit. Many patients have reported finding it convenient to save travel and precious non-dialysis time. It ensures medical supervision for prompt complication management and can enhance dialysis efficacy by increasing muscle blood flow and reducing cardio-pulmonary recycling [96]. Furthermore, it distracts from the mundane dialysis routine, making time go faster and giving value to the overall dialysis experience [76]. Despite these potential benefits, intradialytic exercise is underutilized, likely due to a lack of randomized controlled trials demonstrating its efficacy on clinical outcomes and insufficient education among nephrologists and nurses [76]. Moreover, as underlined by Zoccali et al. [96], intradialytic exercise is often challenging to organize due to space and staff shortages. However, when performing cost-effectiveness analyses, the resources needed for a walking exercise program are just a tiny fraction of those needed for an intradialytic exercise program.

Consequently, a good strategy may involve patients and staff in these exercise programs. Adopting training programs that require minimal staff assistance can be easily accommodated and would not increase nurses’ workload. In addition, long-term adherence can be guaranteed by a multi-modal intra-dialytic exercise training program that incorporates exercise, educational, and motivational components [97, 98].

*Home-based exercise* training could achieve excellent results by promoting greater patient autonomy and improved cost-effectiveness [31]. However, to date, there are no studies demonstrating the superiority of the home-based modality over others [99, 100]. A recent meta-analysis [83] of 12 RCTs sought to respond by comparing the efficacy of home-based exercise versus intradialytic or usual care. It revealed that, in dialysis patients, home-based exercise training for 3–6 months favored physical function and quality of life as assessed by 6MWT and Short Form (36) Health (SF-36) scores, respectively. The Authors proved that home-based exercise training was superior to usual care and equal to training conducted in dialysis units [80]. Indeed, taking into account the most relevant trials, Excite (EXerCise Introduction To Enhance Performance in Dialysis) for home-based [22] and the recent DiaTT (Dialysis Training Therapy) for intradialytic exercise [101], both achieved the same benefits. Home–based exercise has also been shown to maintain the gain in walking distance achieved in the sixth month for up to 36 months. These results indicate that the legacy effect of physical exercise programs described in the general population also occurs in the dialysis population [102].

In summary, each center may choose between intradialytic and home-based exercise training, considering local resources and conditions. In any case, physical exercise programs benefit dialysis patients wherever they are delivered. Additionally, for KTRs and CKD patients on conservative therapy, exercise training could be planned and performed in groups with patients affected by other chronic diseases (such as diabetes mellitus, hypertension, and heart failure) to reduce costs and enhance sustainability.

Regarding the superiority of *group* versus *individual* exercise for CKD patients, more data is needed. However, this remains a promising area for future studies. Notably, exercise during dialysis sessions is frequently conducted in groups. This communal approach may positively influence patients, encouraging them to start their exercise training.

## Exercise training for CKD patients should include aerobic and resistance exercises


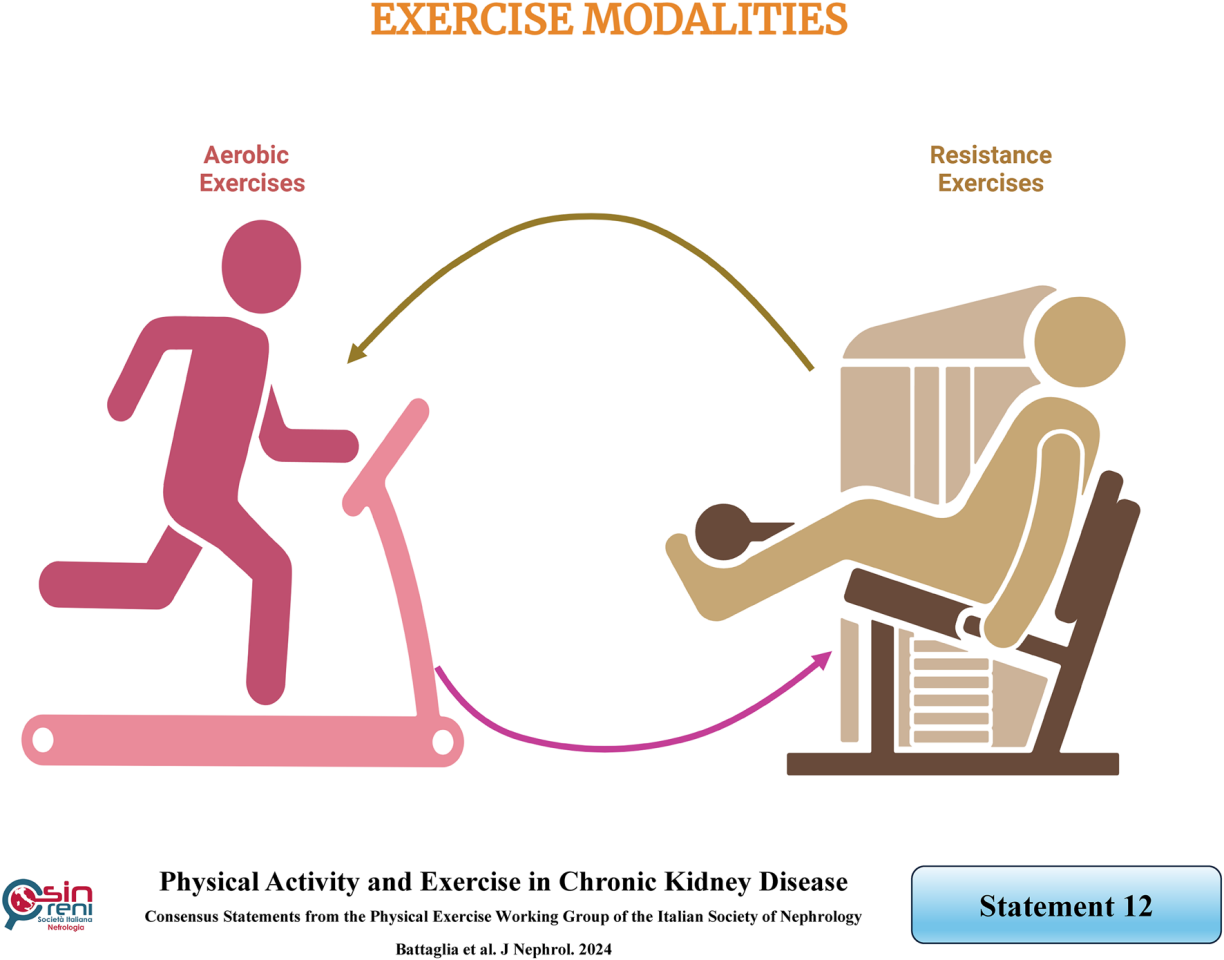


### Rationale

The most effective type of exercise training (i.e., aerobic, strength, combined aerobic and strength, or flexibility routines) has been assessed by several studies in the CKD population (Table 4). Aerobic and resistance exercises may be practicable based on the patient’s needs and requests [103, 104]. However, aerobic exercise stands out as the most often proposed training program [10, 105]. An indirect measurement of aerobic capacity is the maximal oxygen consumption (VO2 peak), which is one of the most studied parameters to assess cardio-respiratory function and tends to improve post-exercise [85, 106]. A more practical parameter for testing aerobic capacity is the 6MWT, which also improves during aerobic exercise. Sheng et al., in their meta-analysis, assessed the effects of different exercise modalities on V02 peak and found that, although still underutilized, the combination of aerobic and resistance exercise is more effective in increasing VO2 peak than solely engaging in aerobic exercise [94]. This finding is likely attributed to the fact that, in some patients, severe muscle atrophy can limit the increase in V02 peak [107].

Some studies propose strength exercise exclusively, sometimes focusing on the lower limbs or incorporating both the lower and upper limbs. In contrast, few studies suggest flexibility exercises [21, 87].

To date, there is still no consensus on the most effective type of exercise to improve physical performance [80], but a combination of both aerobic and resistance exercises can simultaneously provide the advantages of each type [108]. In line with this, a Cochrane systematic review by Heiwe and Jacobson suggested engaging in regular high-intensity mixed cardiovascular and resistance training for at least 30 min per session, three times per week, to achieve the optimal effect on physical fitness, walking capacity, cardiovascular parameters, nutritional status, and health-related quality of life. These beneficial effects have been observed in patients with stage 1–5 CKD, dialysis patients, and KTRs [24].

## Exercise programs should be carried out regularly for at least 12 weeks to be effective


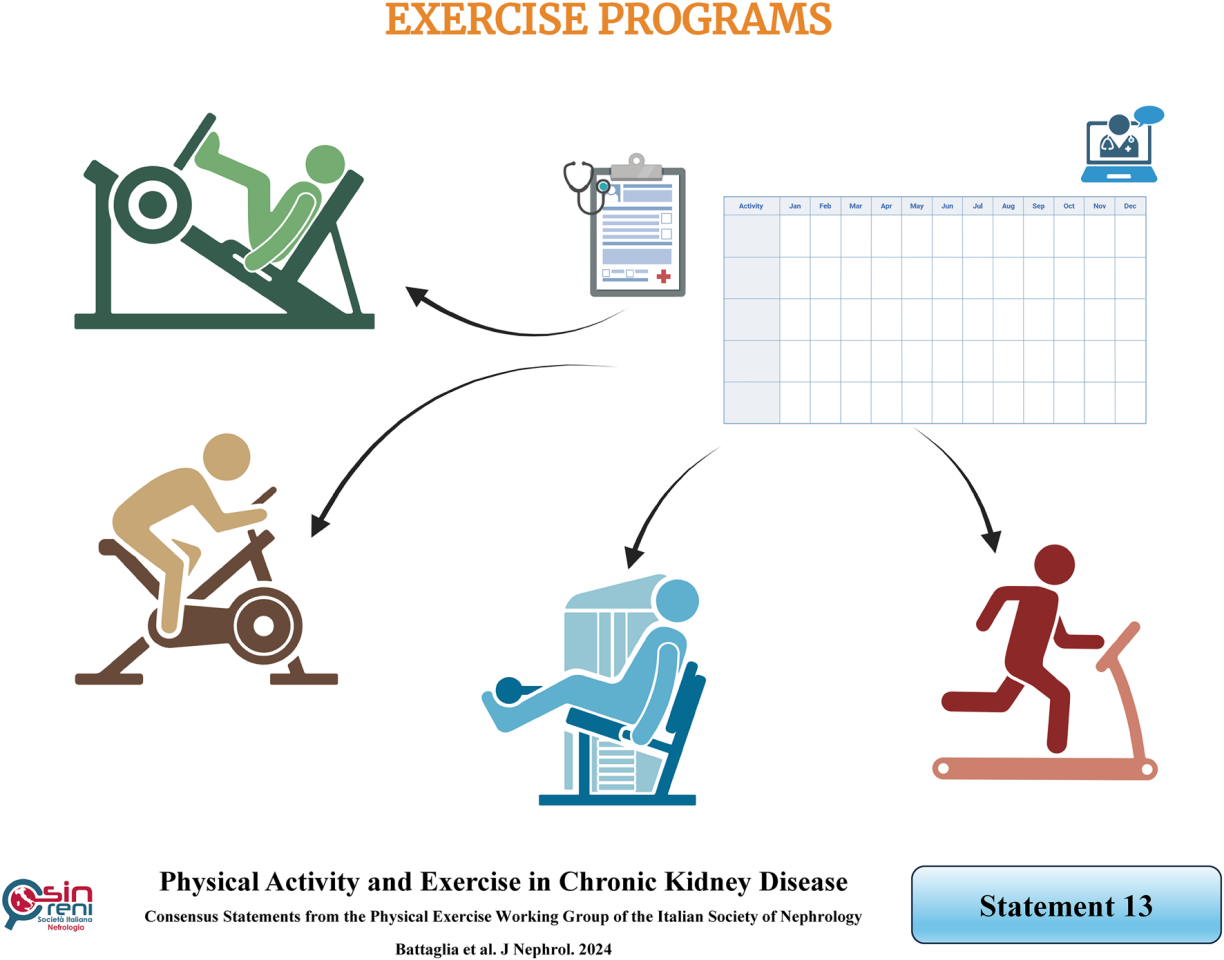


### Rationale

As early as 2005, Stack et al. analyzed the survival advantages of dialysis patients who exercised 2–3 times/week or 4–5 times/week [89]. The duration of treatment proposed in multiple studies ranges from 8 weeks to 12 months, with a prevalence of activity duration of 3 months. In a Cochrane systematic review of forty‐five studies with 1863 participants, the interventions were predominantly short-term (three months) in 17 (37.7%) studies, followed by medium-term (four to six months) in 14 (31.1%) studies and long-term (seven to 12 months) in 14 (31.1%) studies [91]. Exercise training or physical activity lasting 12 months or more is less commonly practiced due to higher dropout rates, although longer treatments may significantly affect structural restoration [88]. In particular, an increase in VO2 peak was observed for exercise training programs longer than six months, even if the effects were minimal [76, 88]. Studies on aerobic exercise showed that variation in duration did not significantly alter the effectiveness of VO2 peak [84, 90]. It is noteworthy that, regardless of the treatment duration, the key is the regularity of exercise training to break sedentary habits in CKD patients. Indeed, regular exercise, irrespective of type, intensity, or intervention length, has consistently demonstrated improvements in aerobic capacity [91]. Further studies with a longer duration of exercise intervention (more than 12 months) should be planned to assess the long‐term benefits of exercise training in CKD patients, such as morbidity and mortality.

## Physical activity and exercise programs should begin with low intensity and progress gradually according to the patient's tolerance


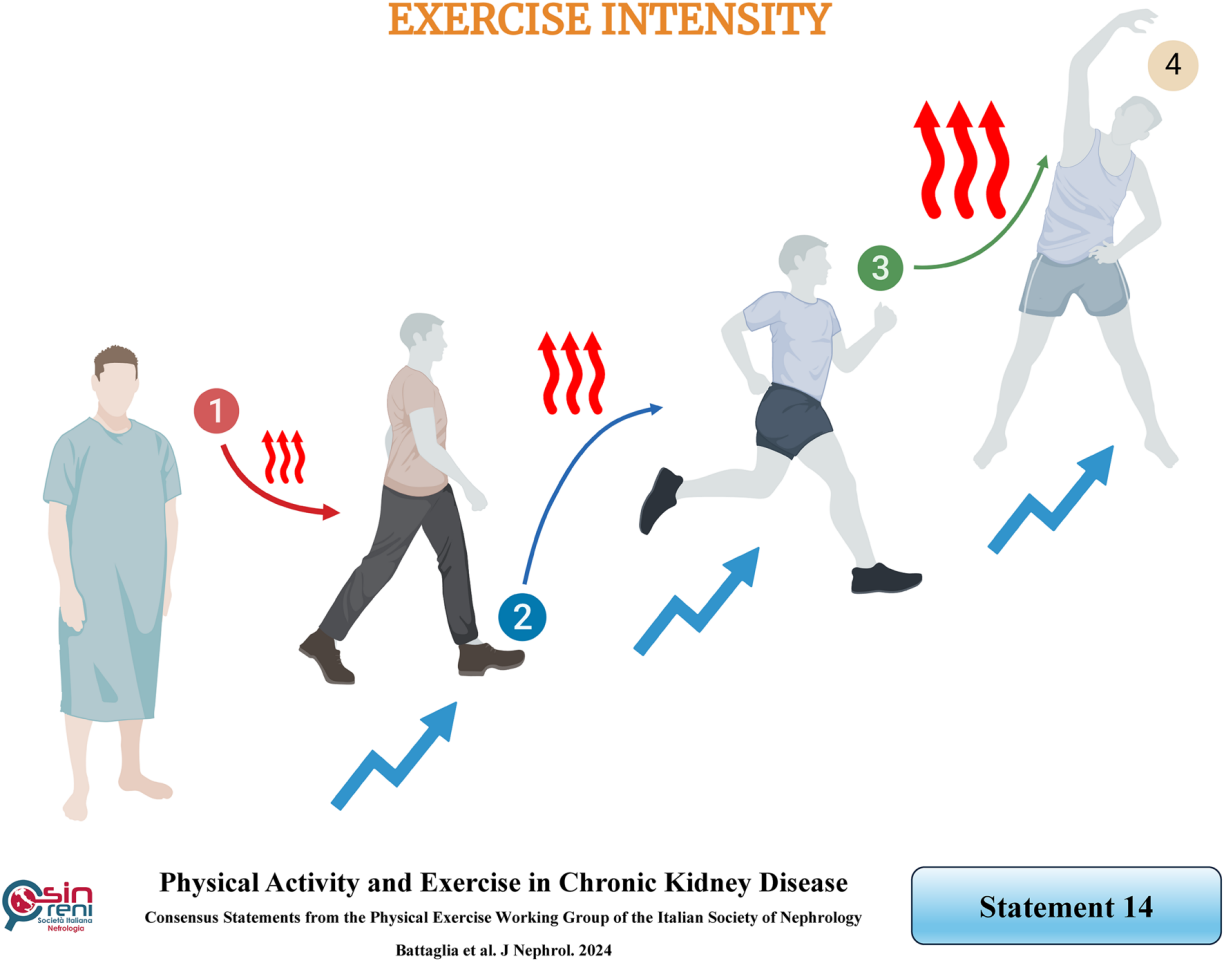


### Rationale

The patient’s tolerance to exercise depends on various elements, encompassing the type, frequency, volume, intensity, duration, and progressive exercise overload. Exercise intensity is critical for ensuring patient compliance. It is a crucial parameter that must be adjusted in a training program based on the patient's prior physical activity to facilitate gradual progression.

The intensity of physical activity is objectively defined by the unit of the metabolic equivalent of task (MET) scores based on the NHANES guidelines. One MET corresponds to the energy consumed at rest. A MET score less than 4 corresponds to light activities, while a score between 4 and 6 represents moderate activity, and a score higher than 6 denotes vigorous activity [109].

According to the United States Physical Activity Guidelines, patients are characterized based on their MET/min/week scores: extremely active if the score is > 1500, very active if > 600 but < 1500, low active if > 0 and < 600, or inactive if 0 [110]. A study exploring the relationship between physical activity and mortality in the general population noted gender differences: for men, there was a reduction in mortality for any level of physical activity, while for women, only extremely intense activity reduced mortality. Therefore, even when considering gender, physical activity should be individualized [111].

However, the exercise prescription guided by the MET score is limited in clinical practice [112], while subjective measurement of exercise intensity is routinely employed. The most common self-reporting method to gauge the intensity of exercise based on the patient’s physical exertion is the rating of perceived exertion (RPE) [113]. It depends on various factors, including heart rate and breathing rate. Subjects' heart rate reserve typically determines the maximum heart rate (HRmax) and maximum oxygen uptake (VOmax).

Currently, the Borg 15-point RPE Scale [76] is the most widely used tool to assess rating of perceived exertion as it is valid, inexpensive, and handy for monitoring individual perceptions of exercise intensity. It ranges from a score of 6, corresponding to no exercise, to 20, corresponding to maximum exercise. A Borg RPE score from 11 to 13 indicates moderate-intensity exercise for CKD patients, while a score of 14 or more indicates vigorous intensity [114].

## In everyday clinical practice, renal rehabilitation should be provided by a healthcare team (nephrologists, nurses, dietitians, and exercise professionals) and, ideally , reimbursed by the national healthcare system


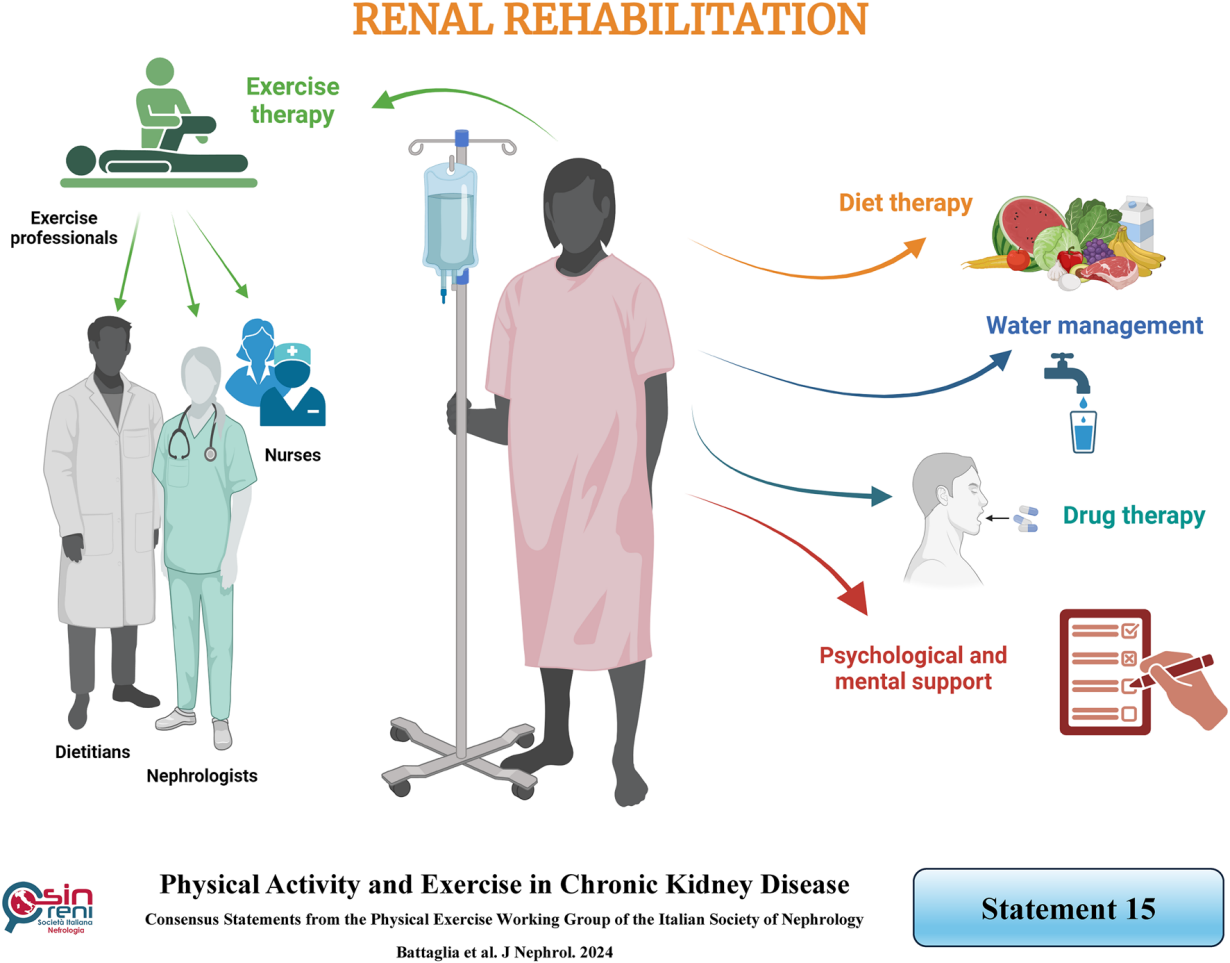


### Rationale

KDIGO and KDOQI guidelines recommend that physicians stimulate CKD patients to engage in daily exercise [1, 115]. Therefore, exercise training should be included in the routine patient care plan, regarded as renal rehabilitation. Specifically, according to the WHO, renal rehabilitation is defined as “a long-term comprehensive program consisting of exercise therapy, diet therapy, water management, drug therapy, physiological and mental support, to alleviate the physical/mental effects based on renal disease and dialysis therapy, prolong life expectancy, and improve psychosocial and occupational circumstances” [116]. Indeed, incorporating renal rehabilitation as a routine treatment in CKD patients’ care plans could reduce all-cause mortality and improve clinical outcomes, quality of life, and life expectancy [4]. In order to assess the sustainability of renal rehabilitation, cost-effectiveness studies with long follow-ups should be conducted. However, currently, rehabilitation therapy involving physical activity or exercise training remains rarely prescribed in patients with CKD. Therefore, one effective strategy is to adopt a multidisciplinary approach involving the entire nephrology healthcare team, which includes nephrologists, nurses, dietitians, and exercise professionals [117].

Among these, *nephrologists* play a central role in improving exercise prescription and addressing sedentary behavior for many reasons [118, 119]. Nephrologists may assess the patient's activity level. They can educate patients about the benefits of exercise and physical activity, providing written information to reinforce them [120]. They can recommend a supportive exercise professional and discuss with the patient, for instance, the feasibility of incorporating physical activity and exercise training during dialysis sessions, utilizing treatment time [68].

*Nurses*, due to their unique relationship with patients and exercise professionals on account of their specific academic training, are often designated as responsible for exercise programs, yielding optimal results [121]. A randomized controlled trial demonstrated the effectiveness of the nurse-led intradialytic exercise program and a home-based exercise program supervised by a physical therapist [79]. Similar results were found in patients with CKD on conservative therapy [22]. In a recent survey conducted by Bennet et al. in peritoneal dialysis patients, clinicians expressed enthusiasm for promoting exercise training with a multidisciplinary team involving different experts in exercise, such as exercise physiologists, kinesiologists, physical therapists [122].

*Dietitians* also play an essential role in prescribing an adequate diet for better physical performance. Indeed, patients with renal dysfunction frequently and precociously suffer from skeletal muscle atrophy. This muscle mass loss is attributed to insufficient nutrient intake or an imbalance between protein synthesis and degradation, especially in dialysis patients [96]. In line with this approach, the Clinical Practice Guidelines for Renal Rehabilitation provide guidance not only for exercise training but also for diet, to be undertaken in conservative, kidney transplanted and dialysis patients [123].

In order to develop and promote this multidisciplinary approach, it is fundamental to consider the possibility of recognizing prescribed physical activity as a therapy reimbursable by the national health care system, a path already embraced by other countries. Currently, Japan is the country furthest along in this regard. In April 2022, Japan's Ministry of Health, Labor, and Welfare became the world's first to extend rehabilitation coverage for intradialytic exercise under the National Health Insurance Reimbursement [124]. Similarly, the Italian Ministry of Health has been recently (as of August 2024) working on a draft law to offer reimbursement for exercise to the entire population.

To summarize, the the presence of an exercise team, the promotion of a culture of exercise, and the increase in physical activity levels lead to a more comprehensive and modern clinical care management of CKD patients [68, 100].

## New technologies, such as interactive social media creation and virtual reality gaming, could improve CKD patients’ adherence to exercise implementation


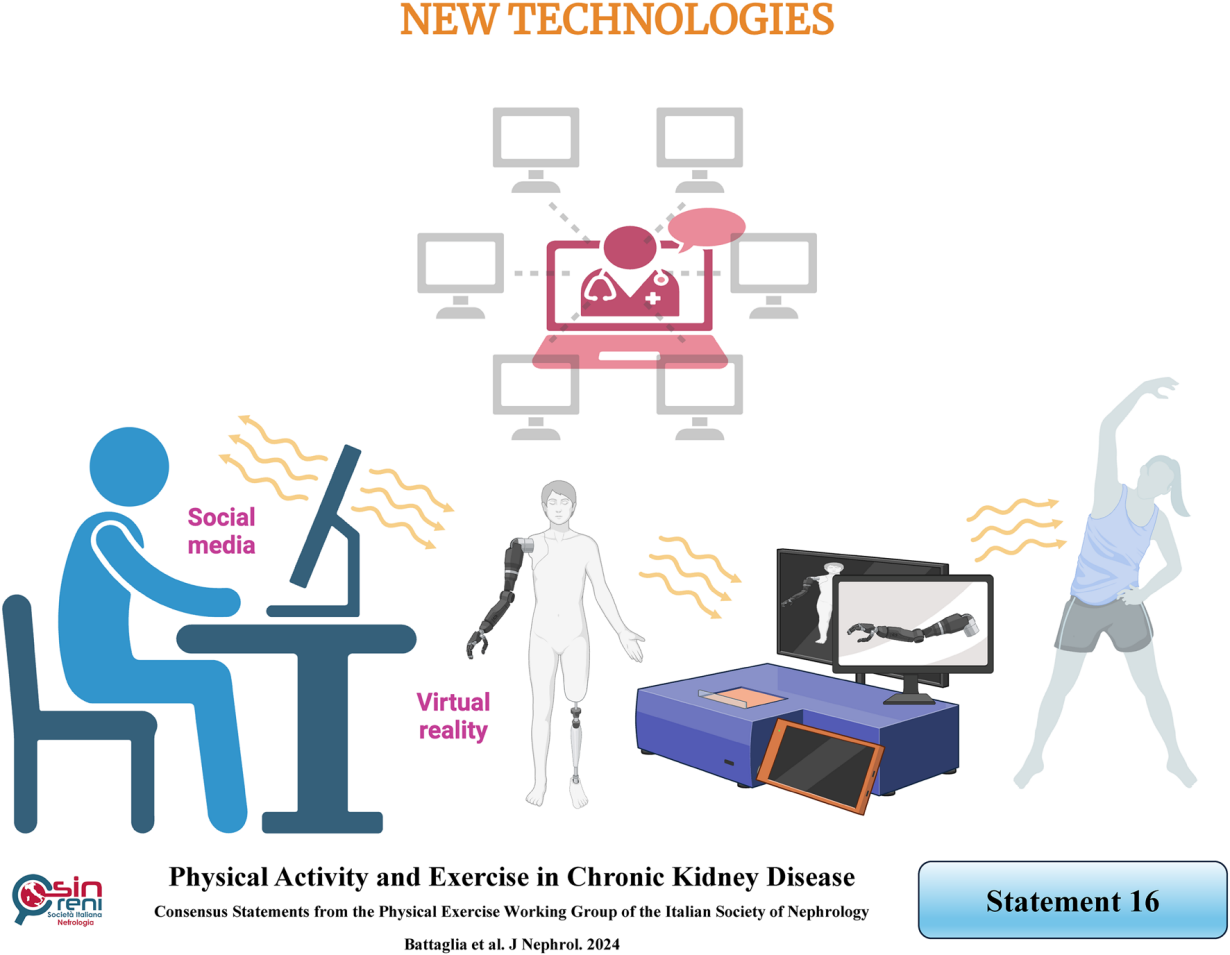


### Rationale

In recent years, technology has opened new perspectives, from the creation of social networks for information dissemination and social interaction to the production of virtual reality gaming, intradialytic yoga, or electrical stimulation of muscles, proposed to enhance exercise programs [125, 126]. However, the literature on this topic needs more evidence.

*Social media*, often associated with health management platforms, has beneficial effects in promoting healthy lifestyle behaviors and inducing changes in exercise habits among CKD patients. The existence of a virtual community could empower patients and improve their physical performance [127].

*Virtual Reality gaming* involves an electronic game with a computer-based system. Players have an immersive 360-degree view in a simulation of a virtual setting using a helmet-mounted display or a flat-screen television. They experience real interaction with the environment while carrying out tasks. This modality allows for engaging in physical activity while playing a game [128]. Virtual reality gaming is typically integrated into intradialytic exercise and appears to be effective in improving physical performance and quality of life, potentially playing a crucial role in adherence [129, 130]. In a scoping review outlining the most common exercise prescriptions, the virtual reality exergame (game combined with exercise) protocol was prescribed during dialysis, in the first half of treatment, for three days per week for four weeks [99].

Few studies have analyzed multicomponent *yoga* in patients with CKD, primarily those on dialysis [131]. Briefly, multicomponent yoga involves movements, breathwork, and meditation. It is a low-intensity exercise program suitable for patients with more compromised cardiovascular frameworks [108]

## Conclusions

The physical exercise working group of the Italian Society of Nephrology has identified sixteen statements aimed at addressing clinically relevant issues in physical activity and exercise for the CKD population. Each point offers practical suggestions to promote a safe approach to facilitate the implementation of physical activity and exercise in clinical practice. The nephrology community should support nephrology and dialysis units to offer effective renal rehabilitation therapy, providing adequate counseling, prescribing appropriate diets, assessing physical performance, and tailoring physical activity prescription, monitoring physical improvements, involving exercise experts, and employing new virtual technologies and social media.

## Data Availability

Not applicable.
